# Comprehensive Phylogenetic Reconstructions of African Swine Fever Virus: Proposal for a New Classification and Molecular Dating of the Virus

**DOI:** 10.1371/journal.pone.0069662

**Published:** 2013-07-25

**Authors:** Vincent Michaud, Tantely Randriamparany, Emmanuel Albina

**Affiliations:** 1 CIRAD, UMR CMAEE, Montpellier, France; 2 CIRAD, UMR CMAEE, Petit-Bourg, Guadeloupe, France; 3 INRA, UMR1309 CMAEE, Montpellier, France; 4 Laboratoire National de Diagnostic Vétérinaire, Antananarivo, Madagascar; Saint Louis University, United States of America

## Abstract

African swine fever (ASF) is a highly lethal disease of domestic pigs caused by the only known DNA arbovirus. It was first described in Kenya in 1921 and since then many isolates have been collected worldwide. However, although several phylogenetic studies have been carried out to understand the relationships between the isolates, no molecular dating analyses have been achieved so far. In this paper, comprehensive phylogenetic reconstructions were made using newly generated, publicly available sequences of hundreds of ASFV isolates from the past 70 years. Analyses focused on B646L, CP204L, and E183L genes from 356, 251, and 123 isolates, respectively. Phylogenetic analyses were achieved using maximum likelihood and Bayesian coalescence methods. A new lineage-based nomenclature is proposed to designate 35 different clusters. In addition, dating of ASFV origin was carried out from the molecular data sets. To avoid bias, diversity due to positive selection or recombination events was neutralized. The molecular clock analyses revealed that ASFV strains currently circulating have evolved over 300 years, with a time to the most recent common ancestor (TMRCA) in the early 18^th^ century.

## Introduction

African swine fever (ASF) is an infectious and contagious hemorrhagic disease of domestic pigs [1]. It is highly lethal, causing up to 100% mortality in naive animals, with devastating effects on pig production and animal trade, and major economic losses in affected countries [2]. First described by Montgomery in 1921 in Kenya [3], ASF has then been observed in most sub-Saharan countries, where it has often become endemic [4]. From Africa, it reached Europe, i.e. Portugal in 1957 and again in 1960, from where it colonized Spain, France, and Belgium. From there, the virus reached Latin America during the 70s–80s. In Europe, ASF remained endemic in the Iberian Peninsula up to the middle of the 90s and the disease is still present in Sardinia [2]. Recently, it has been re-introduced on the borders of Europe, in Georgia in 2007 [5] and then it extended to the Caucasus and Russia [6]. No vaccine is available and disease control is based only on quarantine and animal slaughtering. In this context, its great ability to spread makes the ASF virus one of the most important infectious threats for the domestic pig industry worldwide. African swine fever virus (ASFV) is a large icosahedral and enveloped dsDNA virus; it is the only recognized DNA arbovirus and also the only member of the *Asfarviridae* family and *Asfivirus* genus [7]. However, ASFV shares characteristics with the other members of the *Nucleo-Cytoplasmic Large DNA virus* family [8], suggesting that they all may have had a common ancestor [9], [10].

ASFV is believed to be an ancestral virus of soft tick (*Ornithodoros* genus) [11] infecting wild swine like warthogs (*Phacochoerus fricanus*), bushpigs (*Potamochoerus porcus*), and giant forest hogs (*Hylochoerus meinertzhageni*) with asymptomatic effects. The virus replicates in ticks and is then transmitted to wild swine during blood feeding; wildlife are considered as the natural reservoir of the virus. The virus can persist in ticks for years, even in quiescent ticks waiting for host feeding. The sylvatic cycle of ASFV established between ticks and wild suids can be maintained indefinitely. This cycle allows the maintenance of virus circulation and probably enables the persistence of ancient viruses and the emergence of new variants. At the laboratory level, virus variants were initially characterized by genome size and enzymatic restriction profiles [12]. A high level of variability is observed mainly within the 35 kb at the 3′ end and the 15 kb at the 5′ end of the genome (170–190 kb) [13], [12], [14]. These two regions contain the multigene families (MGF), which vary in number between isolates and enable virus variability by gene homologous recombination. Moreover, variability is also generated by a change in the number of amino-acid repeats in 14 proteins, including the envelope protein p54 encoded by the E183L gene [15]. More recently, gene sequencing and analysis were introduced to increase differentiation between ASFV isolates collected worldwide. The first group [16] used phylogenetic reconstructions based on the partial sequence of B646L gene coding for the major viral protein (MCP) VP72. Their trees showed a very close relationship between West African, European, and South American isolates, all clustered in genotype 1. Despite more than 50 years of circulation in three continents, the limited accumulation of genetic changes has made it impossible to discriminate isolates within genotype 1. In contrast, eastern and southern African isolates are more diverse and segregate into 21 additional genotypes [16], [17], [18]. This could be explained by the fact that these viruses are propagated within a sylvatic cycle, in contrast to viruses of genotype 1 that mainly replicate in domestic pigs, although they were secondarily detected in European soft ticks *O. erraticus* and wild boars in Spain and Portugal. This supports the assumption that the virus diversity may be generated during the sylvatic cycle of the virus [19]. Other genes or genome sequences have been used successfully to discriminate ASFV isolates collected at a regional level. For instance, the B602L gene from the central variable region of the genome (CVR, coding J9L protein), the CP204L gene (coding the phospho-protein P32), and the E183L gene (envelope protein p54) have been used to further split the local isolates [20], [21], [5].

The aim of this study was to reassess the phylogenetic reconstructions and nomenclature of ASFV by including recent sequences and to explore the evolution of the virus based on a comprehensive analysis of the available sequence data sets. Accordingly, three genes were targeted, all of them being the most sequenced and uploaded in public databases. The B646L, E183L, and CP204L genes belong to the most conserved central part of the genome and encode the structural virus proteins VP72 (capsid), p54 (membrane protein), and p32 (membrane protein), respectively. They are also known to generate antibodies in pig [22]. The origin and the evolution of the virus were inferred from these three genes.

## Materials and Methods

### Data Set

A large collection of ASFV isolates were included (Table S1). The majority of ASFV sequences used were downloaded from GenBank (http://www.ncbi.nlm.nih.gov) and a CISA-INIA web site data bank (http://wwwx.inia.es/cisa/asfv/). Additional sequences of Madagascar isolates were generated after virus isolation on pig alveolar macrophages from pigs sampled during outbreaks between 1998 and 2008. These sequences are interesting for the study of ASFV evolution since they are considered to have derived from a unique introduction of the virus in 1998: twenty-one samples were selected to cover the whole territory and the 1998–2008 period. PCRs were performed using the following primers: VP72-d (5′-GGCACAAGTTCGGACATGT-3′) and VP72-U (5′-GTACTGTAACGAAGCAGCACAG-3′) [16], E183L for p54 (5′-GGTTGGTTTTCAAATGTTGGCGAAGGTA-3′) and E183Lrev p54 (5′-CCATAAATTCTGTAATTTCATTGCGCCACAAC-3′), and p30/32-P1 (5′-TG CCAAGCATACATAAGTTG-3′) and p30/32-P2 (5′-ATTT TGCTGTTTATGAATCC-3′) [5] for the amplification of B646L, E183L, and CP204L genes, respectively. PCR products were cloned in *E coli*, and sequences from these clones were generated by a private company (Cogenics, France). Sites with mutations were particularly checked for sequencing errors: only bases confirmed by two-direction reading were retained as mutations. In all, the analyses were performed on 356 sequences (399 nt long), 251 sequences (480 nt long), and 123 sequences (543 nt long) for B646L, E183L, and CP204L genes, respectively.

### Phylogenetic Inference

#### Sequence analysis

Sequences were aligned by ClustalW with default parameters and then scrutinized and edited using Mega version 5 software [23]. From the multiple sequence alignments, an index of substitution saturation to estimate the degree of sequence information was calculated using Dambe software [24]. DNA polymorphism was also analyzed. The site diversity between two sequences (π) and the number of segregating sites (i.e. the number of sites where one or several substitutions occurred) were obtained by DnaSP version 5 software [25]. In the segregating sites, the ratio of transitions and transversions was assessed. The average number of nucleotide differences (k) between two sequences was also determined. All this information was used to check the quality of the sequences.

#### Test for recombination

The presence of sequence recombination events in the data set was assessed in the multiple alignments with RDP3 package version 3 [26] using the default setting for all recombination tests applied on linear sequences (RDP [27], GENECONV [28], MAXCHI [29], BOOTSCAN/RESCAN [30], and SISCAN [31]).

#### Phylogenetic reconstruction

Maximum likelihood reconstructions [32] generating trees that best fit the evolution of a set of sequences through a probabilistic model of evolution were done using TREEFINDER version March 2011 software [33]. The evolution model was selected according to the Akaike information criterion (AIC) [34], the corrected AIC (AICc) [35], and Bayesian Information Criterion (BIC) [36] with a number of gamma rate categories fixed at 5. The consensus model given by the three information criteria or alternatively, the simplest model, was selected for the reconstruction. Thus, the B646L tree was constructed under HKY+Г_5_ model [37], [38]. For E183L and CP204L, HKY+Г_5_ and HKY on the one hand and HKY+Г_5_ and TN+ Г_5_ models on the other hand were selected. The most complex model, GTR [39], was also systematically included and compared with the others. All the reconstructions were done on 1,000 replicates and bootstraps were approximated using the Expected-Likelihood Weights defined by Strimmer and Rambaut (2002) [40] applied on local rearrangements (LR-ELW) as implemented in TREEFINDER.

Bayesian inference phylogeny was performed using Monte Carlo Markov Chain (MCMC) implemented in MrBayes version 3.1 software [41], [42]. According to the best fit models proposed by TREEFINDER, MrBayes was set with HKY+Г_5_, HKY and HKY+Г_5_, and HKY+ Г_5_ for B646L, E183L, and CP204L, respectively. The GTR model was also used for each gene. MCMC was run for a maximum of 10 million trees or alternatively when the run reached stationarity as measured by a standard deviation of split frequencies either becoming lower than 0.01 or fluctuating randomly above 0.01 for at least 500,000 generated trees. Consensus trees were generated after having discarded the first 25% of the MCMC burn-in phase.

Tree congruence with data sets was tested by submitting them to the statistical test ELW [40] implemented in TREEFINDER. The tree selected for each gene was the one with the highest ELW score.

Since ASFV is the only member of the *Asfarviridae* family, 37 outgroup viruses for tree rooting were selected in the closest related DNA virus families, the NCLDVs [43], [44], [45]. Because of the high level of nucleotide divergence, multiple sequence alignments were done on the complete amino-acid sequences of the major capsid protein of both outgroup viruses and ASFV isolates (equivalent to B646L protein) using Mega5 software (see File S1). Tree reconstructions were performed on 1,000 replicates using maximum likelihood method set with WAG+G+I+F and WAG+G+I models using “all sites” and “complete deletion” options, respectively. The topology of the resulting rooted tree was subsequently applied for placing roots on the B646L, E183L, and CP204L trees.

#### Analysis of selection pressure

Codons under positive selection pressure in DNA coding sequences may evolve faster than the natural evolutionary rate of the virus genome. To avoid bias in the molecular clocking analysis, the selection pressure acting on the targeted genes was assessed. The ratio of non-synonymous (dN) – synonymous substitution (dS) per site (dN/dS ratio) was calculated and the codons under positive selection pressure were identified by using Codeml software implemented in PAML 4 package.

#### ASFV genotyping

Isolate genotyping was assessed by comparing the genetic distance between all B646L sequences. Average intra- and inter-branch distances were globally compared to determine the strength of cluster segregation. Additionally, a haplotype network of the isolates was constructed using TCS1.21 software to identify relationships between isolates potentially poorly represented by conventional phylogenetic tree reconstruction. Lastly, specific nucleotide signatures of the different ASFV clusters were searched using multiple sequence alignments containing only the 67 unique B646L sequences. The three approaches were finally combined to raise conclusions about ASFV genotyping.

#### Molecular dating

Two methods were used in parallel and compared to determine the evolutionary rate and the time to the most recent common ancestor (TMRCA) of circulating ASFV isolates. The first was based on the maximum likelihood method Baseml implemented in PAML 4 package [46] and the second on the Bayesian MCMC implemented in BEAST package version 1.6.2 [47]. Codons under positive selection (dN/dS >1) and recombined sequences were removed from the multiple sequence alignments to avoid bias in the substitution rate determination and consequently in the Tmrca estimation. The best fit tree generated in the phylogenetic reconstructions was used to perform Baseml implemented in PAML 4 package, using as evolution model HKY+Г_5_ for B646L, CP204L, and E183L genes. Strict and relaxed molecular clock hypotheses [48] were used to generate dated trees for all genes. These two trees were individually compared with the tree generated without a clock constraint to accept or reject the molecular clock hypothesis. A likelihood ratio test (LRT) and a χ^2^ comparison were performed to support this analysis. For the relaxed molecular clock, branches delineating the different genotypes were individually relaxed.

All analyses performed with BEAST package were done under an uncorrelated lognormal relaxed clock model. Considering that at least 20% of our sequences were from isolates persisting in wildlife, a constant population size prior was selected. The initial value and the range of substitution rates were estimated from preliminary analyses and entered into the model of evolution. For each gene, analyses of two independent runs of 100 million steps were performed with 1/10,000 trees sampled. MCMC samples were examined using Tracer version 1.4 [49]; the first 25% of samples in the chain were discarded as burn-in phase. Tree consensus was generated using the maximum clade credibility (MCC) tree using Tree Annotator version 1.4.7 [47]. Only posterior probabilities higher than 0.90 are indicated.

#### Tree visualization

All trees were represented and edited in Fig Tree version 1.3.1 developed by Andrew Rambaut (http://tree.bio.ed.ac.uk/software/figtree/).

## Results

### Comprehensive Phylogenetic Inference of ASFV Depicts 4 Major Lineages

**Figure 1:**
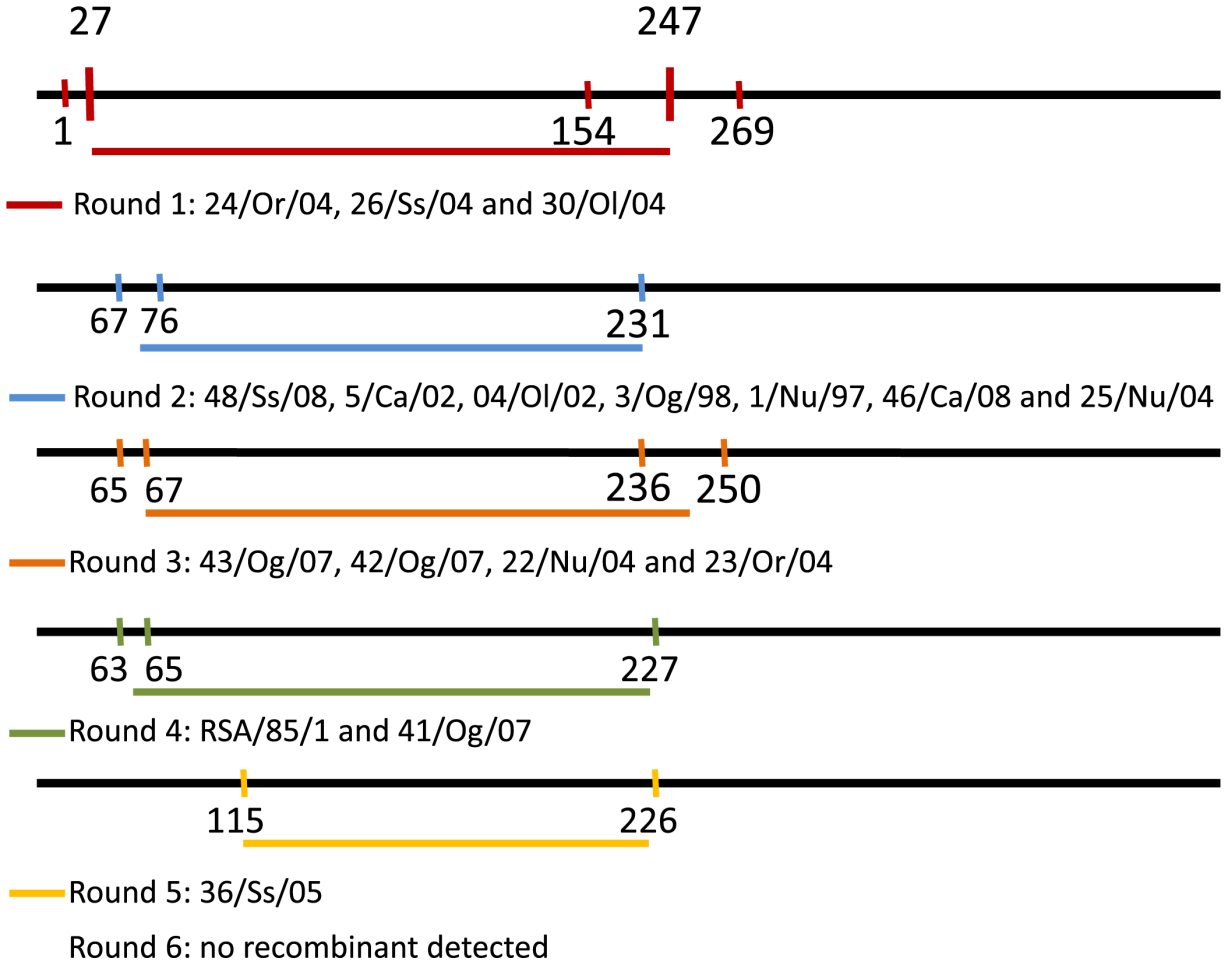
Localization of recombination events detected in E183L sequence alignment. 16 Italian isolates and 1 South African isolate were detected to be recombinant. Italian isolates are linked and because recombination events take place in the same region of the sequence, these isolates have probably emerged from a common ancestor.

Before phylogenetic inference, data sets and multiple sequence alignments were thoroughly examined to eliminate misalignments and ensure correct framing of coding sequences. All gaps were considered as missing information to avoid artificial nucleotide divergence. None of the different methods used in RDP3 package identified recombination events in B646L and CP204L sequences. In contrast, several recombination events were detected among E183L sequences. A total of 17 isolates were subsequently removed from the E183L multiple sequence alignments: 16 were Italian isolates (24/Or/04, 26/Ss/04, 30/Ol/04, 48/Ss/08, 5/Ca/02, 04/Ol/02, 3/Og/98, 1/Nu/97, 46/Ca/08, 25/Nu/04, 43/Og/07, 42/Og/0, 22/Nu/04, 23/Or/04, 41/Og/07 and 36/Ss/05) [50] and one was a South African isolate (RSA/85/1). The recombination events were all identical for Italian isolates (Figure 1). There were no saturated codons in our alignments (DAMBE, p_value_<<0.03), thus indicating the genetic information in the data sets was suitable for phylogenetic studies.

To check the nucleotide composition of the alignments, statistical tests were performed using DnaSP software. The tests gave the number of nucleotide substitutions, the average diversity per site between two sequences (π), and the average nucleotide difference between two sequences (k). The diversity of B646L and CP204L was approximately half that of E183L (Table 1). In addition, E183L showed a clear bias in non-synonymous mutations. Based on the observed nucleotide substitutions, the minimum and the maximum evolutionary rates were also calculated from each multiple alignment (Table 1). We determined the *dN/dS* of each gene (Table 1) and the amino acids under positive selection in the alignments. This led to removing 6 nt (2 aa: His4 and Thr28) from B646L alignments, 9 nt (3 aa: Glu31, Pro123 and Leu176)) from CP204L alignments, and 27 nt (9 aa: Tyr10, Thr23, Asp100, Thr104, Ser122, Pro140, Val142, Glu143 and Ser149) from E183L alignments for subsequent molecular dating analyses.

**Table 1:** Summary of the multiple sequence alignments analyses.

Gene	Size, in nt	Polymorphic sites	Syn.	Non Syn.	π	k	dN/dS
B646L	399	110 (27.6%)	62	60	0.03416	13.629	0.223
B646L w/o P	393	–	–	–	–	–	–
E183L	480	263 (54.7%)	88	214	0.07283	34.960	0.286
E183L w/o P	453	–	–	–	–	–	–
CP204L	543	154 (28.2%)	83	88	0.06738	36.588	1.123
CP204L w/o P	534	–	–	–	–	–	–

π: average diversity per site between two sequences (number of nucleotide differences per site between two sequences). k: average number of nucleotide differences between two sequences. w/o P: without codons under positive selection.

**Figure 2:**
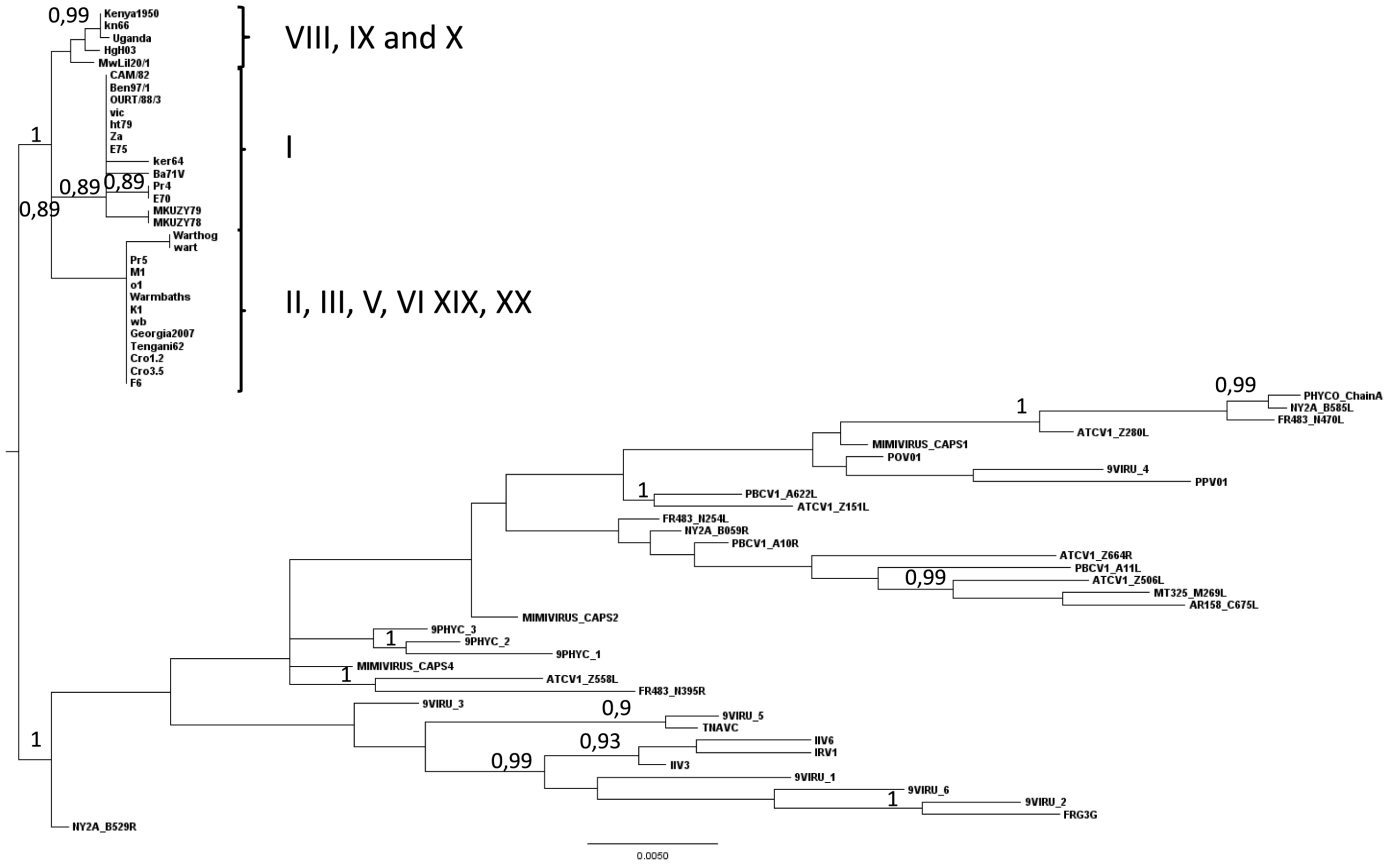
Rooted tree constructed from amino-acid multiple alignments of the major capsid protein of ASFV isolates and four out-grouped viruses. The tree was constructed under a WAG+G+I+F model and maximum likelihood method with 1,000 bootstrap resampling. Numbers indicate the statistical value (Expected-Likelihood Weight) of internal nodes, given in percentages (only numbers above 70% are indicated). The outgroup connects ASFV group by the branch from genotypes VIII (MwLil20/1), IX (UgH03), and X (Kenya1950, kn66 and Uganda) to other genotypes.

The outgroup-rooted trees constructed from the multiple sequence alignments of the major capsid protein amino acid sequences of 30 ASFVs and with 37 out-group viruses from the NCLDV family showed that the common ancestor of all these viruses connects the ASFV group within eastern African isolates, more precisely between the genotype VIII, IX and X on the one hand and genotypes I and the other genotypes on the other hand (Figure 2). Accordingly, the root on all subsequent trees was placed in this position. This reconstruction also shows that the *Asfarviridae* family is rather divergent from the other NCLDV families.

**Figure 3:**
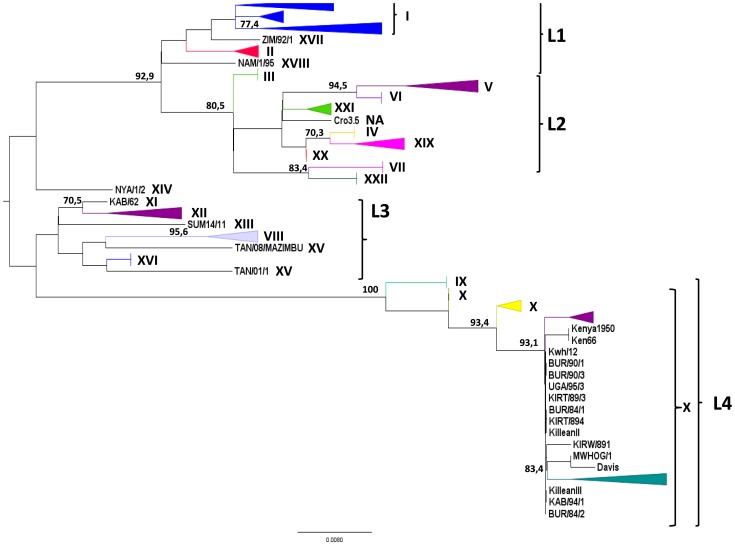
B646L gene phylogenetic tree describing ASFV relationships. The tree was constructed under HKY85+ evolutionary model with 1,000 bootstrap resampling. Numbers indicate the statistical value (Expected-Likelihood Weight) of internal nodes, given in percentages (only numbers higher than 70 are indicated). Lineages were collapsed for improved tree visibility. The tree shows four main lineages (L1 to L4).

**Figure 4:**
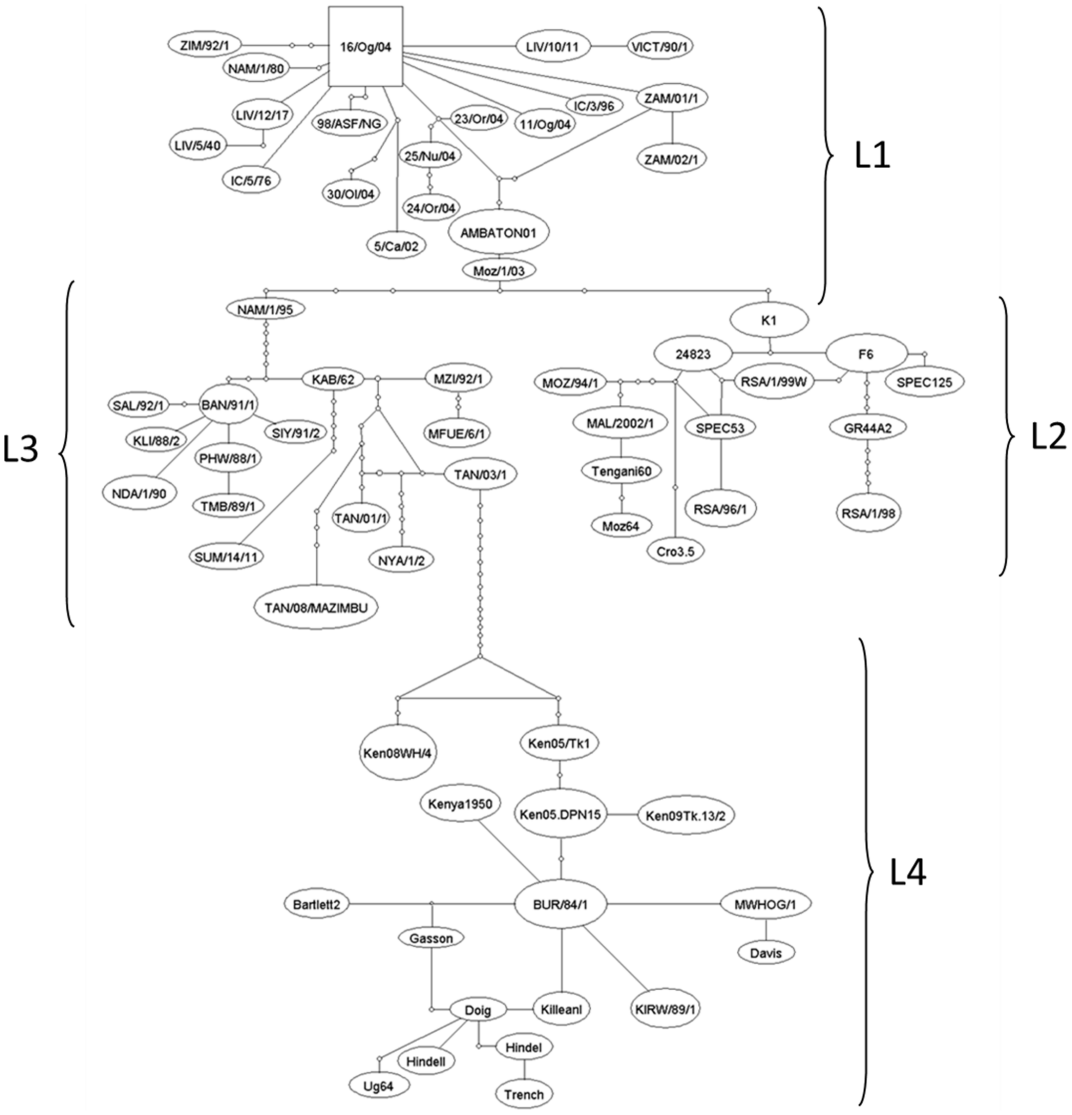
Haplotype network constructed with TCS software. The network shows the same four main lineages that were observed in the bifurcative phylogenetic tree constructed in maximum likelihood under the HKY+ model, but clearly demonstrates that relationships between some ASFV isolates are too complex to be resolved by only bifurcations.

**Figure 5:**
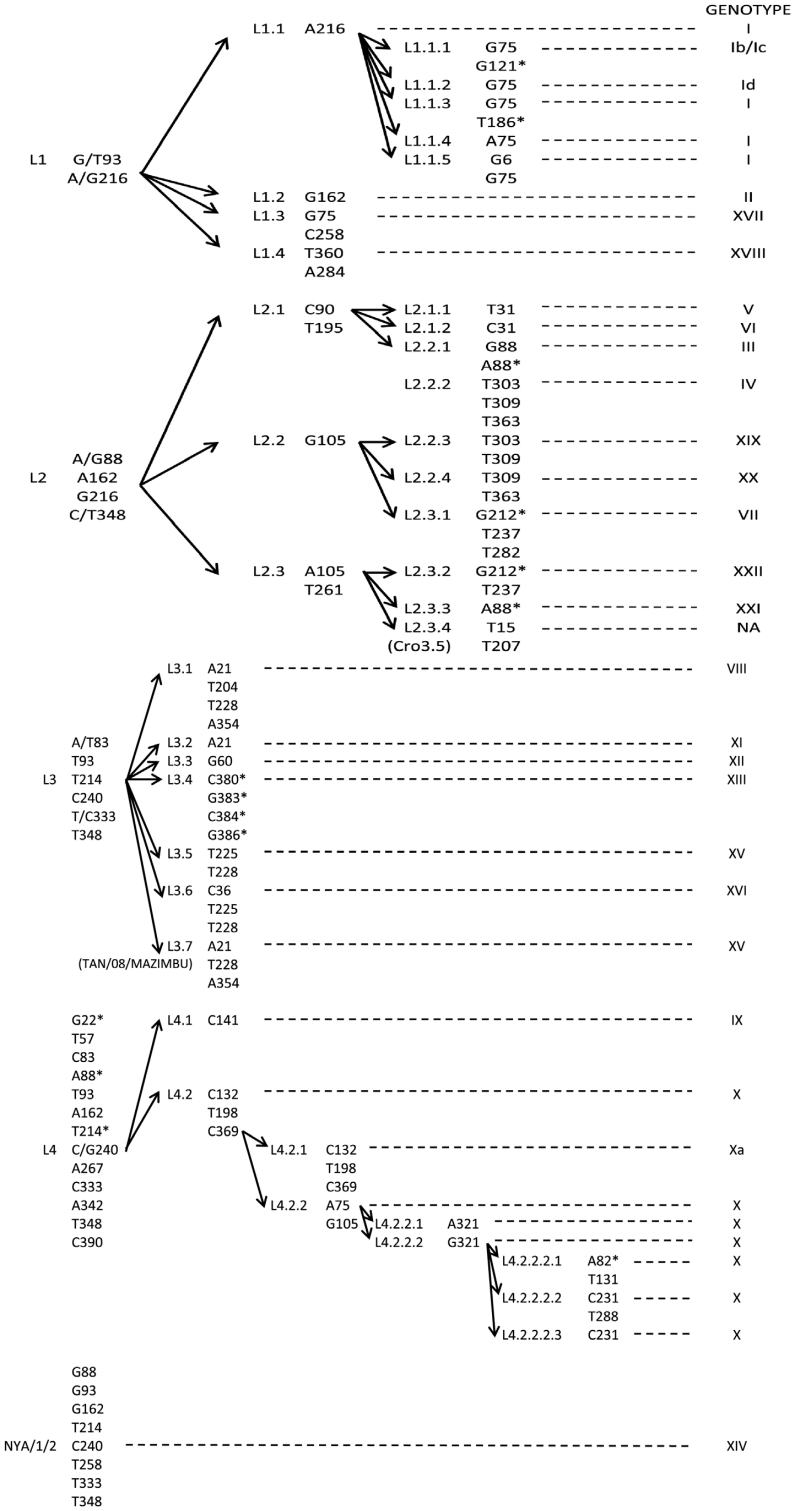
Molecular signatures of ASFV lineages and sub-lineages. Corresponding genotypes are indicated in the right column. Non synonymous substitutions are labeled with “*”. NA: non assigned.

Phylogenetic trees constructed with B646L sequences showed four major lineages (L) (Figure 3): L1 includes the previously described genotypes I, II, XVII, and XVIII, and L2, genotypes III, IV, V, VI, VII, XIX, XX, XXI, and XXII and an ungenotyped isolate (Cro3.5) [51]. L3 includes genotypes VIII, XI, XII, XIII, XV, and XVI and one isolate TAN/08/MAZIMBU, previously included within genotype XV [52]. L4 gathers genotypes IX and X. Interestingly, the NYA/1/2 isolate ascribed to genotype XIV is the only isolate that does not segregate within one of the four lineages. However, the bootstrap value of its branch is <70%, thus rendering difficult any conclusion about this isolate. Further clustering of the isolates within these four lineages becomes tricky because of the presence of long branches and multifurcation for some isolate groups or sub-lineages. The TCS network analysis showed that conventional phylogenetic reconstruction based on bifurcations may fail to explain the complex relationships between some isolates (Figure 4). The TCS network confirms the existence of the four lineages that include the same isolates as in bifurcated reconstructions. However, the TCS network seems to better explain the relationships of isolates within a given genotype (e.g. genotype I or X) or between distinct genotypes (e.g. between genotypes III, IV, XIX, XX, and XXI, or between genotypes IX and X). In these cases the pattern of isolate relationships is not strictly bifurcative. Three ways exist between genotype XIX and genotype XX: through genotypes III or IV and/or XXI, which represent internal nodes of the tree, and two ways between genotypes IX and X. Within genotype X, several isolates are internal nodes of the tree, meaning that an isolate can have more than one ancestor, which is inconsistent with bifurcative relationships between isolates. In attempts to refine the clusterization of ASFV isolates, the multiple sequence alignments containing 67 unique B646L sequences were searched for specific molecular signatures (Figure 5). Lineage 1 is characterized by 2 nt, and L2, L3, and L4 by 4, 6, and 12 nt, respectively. Genotype XIV, which is not included in one of the four lineages, is characterized by 8 nt (G88, G93, G162, T214, C240, T258, T333, and T348). However, this is the only virus generating this branch, which in addition is not supported by a high bootstrap value (<70%). Therefore, it cannot yet be considered as a fifth lineage. Lineages can be subsequently sub-divided into sub-lineages: 4 for lineage 1, 3 for lineage 2, 7 for lineage 3, and 2 for lineage 4 (Figure 5). Further sub-divisions can be drawn from the molecular signatures (Figure 5) and all are supported by the evolutionary distance matrix, except for some sub-lineages within L1-1, L1-2, L1-3, L2-2, L2-3, and L4-2-2 (Table 2). The average evolutionary distances inside and between all sub-lineages were 0.0023 and 0.055, respectively. L4 is the most complex lineage, composed of isolates from countries of the Great Lakes Region in Africa (Tanzania, Uganda, Burundi, and Kenya) and divided into several sub-lineages. Sub-lineage L4.2 (including isolates belonging to former genotype X) is the most diverse, with isolates clustering into seven sub-lineages (from L4-2-1 to L4-2-2-2-3). This new clusterization into lineages almost perfectly overlays the previous genotype discrimination, with the exception of Cro3.5 isolate, which forms a new cluster within L2 (sub-lineage L2-3-4) and TAN/O8/MAZIMBU isolate, which splits from genotype XV to form a new sub-lineage of L3 (L3-7).

The trees generated with CP204L and E183L genes (data not shown) confirmed the existence of four lineages including the same genotypes. However, the E183L gene tree shows some differences in the clustering: SPEC/205 belonged to L1.1.1 lineage with B646L while it moves to L3.2.2.3 (genotype XI) with E183L. NYA/1/2, the sole member of former genotype XIV in B646L classification and which segregated between lineages L1, L2, and L3, is placed within lineage L3 in E183L classification. Whether these modifications may be ascribed to inter-gene recombination events remains unclear.

**Table 2:** Estimates of evolutionary distances between ASF lineages and sub-lineages.

	L1-1	L1-1-1	L1-1-2	L1-1-3	L1-1-4	L1-1-5	L1-2	L1-3	L1-4	L2-1	L2-2	L2-2-1	L2-2-2	L2-2-3	L2-2-4	L2-3-1	L2-3-2	L2-3-3
L1-1	0.000363																	
L1-1-1	0.003126	0.000739																
L1-1-2	0.003845	0.006608	0.002125															
L1-1-3	0.008001	0.010764	0.011483	0.01051														
L1-1-4	0.003247	0.006009	0.006729	0.010885	0.000934													
L1-1-5	0.009782	0.012545	0.013265	0.01742	0.012667	0.010467												
L1-2	0.010783	0.013545	0.014265	0.018421	0.013582	0.020202	0.000102											
L1-3	0.008052	0.010814	0.011534	0.01569	0.010851	0.017471	0.013212	0										
L1-4	0.015949	0.018712	0.019431	0.023587	0.018749	0.025369	0.015766	0.018379	0									
L2-1	0.037955	0.040717	0.041437	0.045593	0.040754	0.047375	0.037772	0.040385	0.037743	0.004706								
L2-2	0.031679	0.034441	0.035161	0.039317	0.034478	0.041099	0.031496	0.034109	0.031467	0.011502	1.66E-15							
L2-2-1	0.018508	0.02127	0.02199	0.026146	0.021307	0.027928	0.018325	0.020938	0.018296	0.024702	0.018425	2.4E-16						
L2-2-2	0.02885	0.031612	0.032331	0.036488	0.031649	0.038269	0.028667	0.031279	0.028637	0.02467	0.018393	0.015596	2.63E-16					
L2-2-3	0.029477	0.032239	0.032959	0.037115	0.032276	0.038897	0.029294	0.031907	0.029265	0.025297	0.019021	0.016223	0.005857	0.001623				
L2-2-4	0.023637	0.0264	0.027119	0.031275	0.026437	0.033057	0.023454	0.026067	0.023425	0.019457	0.013181	0.010384	0.005226	0.005854	1.49E-05			
L2-3-1	0.031783	0.034546	0.035265	0.039421	0.034583	0.041203	0.0316	0.034213	0.031571	0.037973	0.031697	0.01853	0.028867	0.029495	0.023655	1.2E-16		
L2-3-2	0.029087	0.031849	0.032569	0.036725	0.031886	0.038506	0.028904	0.031517	0.028874	0.035277	0.029	0.015833	0.026171	0.026798	0.020959	0.013165	4.17E-15	
L2-3-3	0.025019	0.027782	0.028501	0.032658	0.027819	0.034439	0.024836	0.027449	0.024807	0.020642	0.014366	0.011766	0.011734	0.012361	0.006522	0.025037	0.022341	0.002575
L2-3-4	0.026332	0.029094	0.029814	0.03397	0.029131	0.035751	0.026149	0.028762	0.026119	0.022073	0.015797	0.013078	0.013046	0.013674	0.007834	0.02635	0.023653	0.009137
NYA/1/2	0.029578	0.03234	0.03306	0.037216	0.032377	0.038997	0.029395	0.032007	0.029365	0.051292	0.045016	0.031845	0.042186	0.042814	0.036974	0.04512	0.042424	0.038356
L3-1	0.045886	0.048648	0.049367	0.053524	0.048685	0.055305	0.045703	0.048315	0.045673	0.0676	0.061324	0.048153	0.058494	0.059122	0.053282	0.061428	0.058732	0.054664
L3-2	0.034669	0.037431	0.038151	0.042307	0.037468	0.044088	0.034486	0.037099	0.034456	0.056383	0.050107	0.036936	0.047278	0.047905	0.042065	0.050211	0.047515	0.043448
L3-3	0.038622	0.041384	0.042104	0.04626	0.041421	0.048041	0.038439	0.041051	0.038409	0.060336	0.05406	0.040889	0.05123	0.051858	0.046018	0.054164	0.051468	0.0474
L3-4	0.042908	0.04567	0.04639	0.050546	0.045708	0.052328	0.042725	0.045338	0.042696	0.064623	0.058346	0.045176	0.055517	0.056145	0.050305	0.058451	0.055754	0.051687
L3-5	0.048061	0.050823	0.051543	0.055699	0.05086	0.057481	0.047878	0.050491	0.047849	0.069775	0.063499	0.050328	0.06067	0.061297	0.055458	0.063604	0.060907	0.05684
L3-6	0.037262	0.040024	0.040744	0.0449	0.040061	0.046681	0.037079	0.039692	0.037049	0.058976	0.0527	0.039529	0.04987	0.050498	0.044658	0.052804	0.050108	0.04604
L3-7	0.048091	0.050853	0.051572	0.055729	0.05089	0.05751	0.047908	0.05052	0.047878	0.069805	0.063529	0.050358	0.060699	0.061327	0.055487	0.063633	0.060937	0.056869
L4-1	0.069975	0.072737	0.073456	0.077613	0.072774	0.079394	0.069791	0.072404	0.069762	0.091689	0.085413	0.072242	0.082583	0.083211	0.077371	0.085517	0.082821	0.078753
L4-2-1	0.070095	0.072857	0.073577	0.077733	0.072895	0.079515	0.069912	0.072525	0.069883	0.09181	0.085533	0.072363	0.082704	0.083331	0.077492	0.085638	0.082941	0.078874
L4-2-2-1	0.076065	0.078827	0.079547	0.083703	0.078864	0.085485	0.075882	0.078495	0.075853	0.09778	0.091503	0.078333	0.088674	0.089301	0.083462	0.091608	0.088911	0.084844
L4-2-2-2-1	0.085652	0.088415	0.089134	0.09329	0.088452	0.095072	0.085469	0.088082	0.08544	0.107367	0.101091	0.08792	0.098261	0.098889	0.093049	0.101195	0.098498	0.094431
L4-2-2-2-2	0.089035	0.091797	0.092517	0.096673	0.091835	0.098455	0.088852	0.091465	0.088823	0.11075	0.104473	0.091303	0.101644	0.102272	0.096432	0.104578	0.101881	0.097814
L4-2-2-2-3	0.081494	0.084257	0.084976	0.089132	0.084294	0.090914	0.081311	0.083924	0.081282	0.103209	0.096933	0.083762	0.094103	0.094731	0.088891	0.097037	0.09434	0.090273

L2-3-4	NYA/1/2	L3-1	L3-2	L3-3	L3-4	L3-5	L3-6	L3-7	L4-1	L4-2-1	L4-2-2-1	L4-2-2-2-1	L4-2-2-2-2	L4-2-2-2-3
														
														
														
														
														
														
														
														
														
														
														
														
														
														
														
														
														
														
0														
0.039669	0													
0.055976	0.032641	0.001564												
0.04476	0.021424	0.02158	0											
0.048713	0.025377	0.025532	0.009117	0.007923										
0.052999	0.029664	0.029819	0.01857	0.022523	0									
0.058152	0.034816	0.029775	0.023755	0.027708	0.031995	0								
0.047353	0.024017	0.018975	0.012956	0.016909	0.021195	0.015991	0							
0.058182	0.034846	0.024725	0.023785	0.027737	0.032024	0.03198	0.02118	0						
0.080065	0.05673	0.061858	0.050641	0.054594	0.05888	0.064033	0.053234	0.064063	1.22E-15					
0.080186	0.056851	0.061978	0.050762	0.054715	0.059001	0.064154	0.053355	0.064184	0.013198	4.64E-16				
0.086156	0.062821	0.067948	0.056732	0.060685	0.064971	0.070124	0.059325	0.070154	0.019168	0.006026	0.001692			
0.095743	0.072408	0.077536	0.066319	0.070272	0.074558	0.079711	0.068912	0.079741	0.028755	0.015613	0.011175	0.005192		
0.099126	0.075791	0.080919	0.069702	0.073655	0.077941	0.083094	0.072295	0.083124	0.032138	0.018996	0.014557	0.013714	0.007739	
0.091585	0.06825	0.073378	0.062161	0.066114	0.0704	0.075553	0.064754	0.075583	0.024597	0.011455	0.007017	0.006173	0.009535	0.001909

The average intra-sub-lineage diversity was 0.0023, whereas the average inter-sub-lineage was 0.055. In the matrix, diversities lesser than 5x(intra-sub-lineage diversity) =  0.0115 are shown in grey boxes: all sub-lineages (Lx-x) differ from the others by a higher diversity, except for some sub-lineages within L1.1, L1.2, L1.3, L2.2, L2.3, and L4.2.2.

### Molecular Dating Leads to a most Recent Common Ancestor of about 300 Years

**Figure 6:**
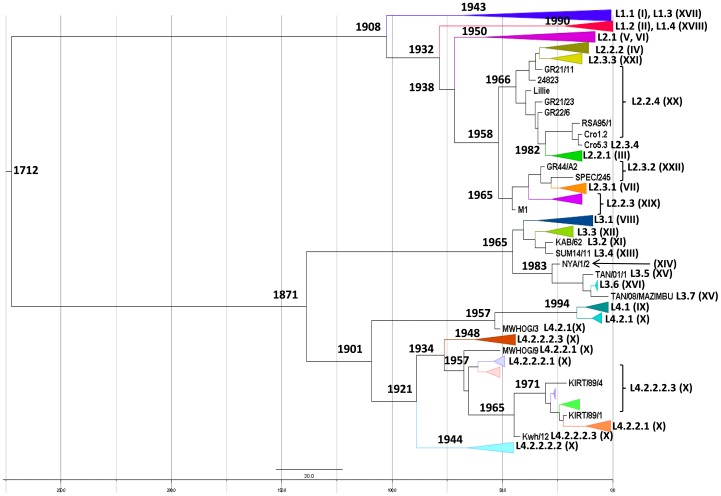
Dated tree representing evolutionary relationships of B646L gene between ASFV isolates. Lineages and corresponding genotypes are indicated. The tree was constructed by Beast software and the MCMC were run 10^8^ times. Time for all isolatesTMRCA is 1712. TMRCAs of lineage L1-1 (genotype I) and lineage L1-2 (genotype II) are 1943 and 1990, respectively.

E183L gene was removed from molecular dating analyses because of the detection of several recombination events and a non-synonymous bias in the gene alignment both due to a strong positive selection of the immune system on this gene. The strict molecular clock hypothesis, meaning an equal substitution rate for every nucleotide site along the DNA sequences, was rejected for the other two genes by the maximum likelihood analysis performed by Baseml in PAML software suite. In PAML, the branches were individually relaxed in the tree submitted to the analysis. Several trees with different numbers of relaxed branches were tested. The resulting TMRCAs for B646L and CP204L genes were highly variable: from 1597 BC to 700 AD or even undetermined date (because of a tree likelihood value of zero at the beginning of the analysis). This high level of heterogeneity in the TMRCA using maximum likelihood method led us to select Bayesian approach in the BEAST package. The Bayesian MCMC inference of the two data sets performed with BEAST package showed a satisfactory convergence in the posterior statistic estimates of the substitution rate. Preliminary analyses were used to set the initial value of µ, the parameter of substitution/site/year (data not shown). Accordingly, the prior distribution of this parameter was set from 0.1× µ to 1× µ. Thus, calibrations of molecular clocks were set at 5.3×10^−3^ substitution/site/year [5.3×10^−4^–1.4×10^−1^] for B646L gene and 5.36×10^−3^ [5.36×10^−4^–1.99×10^−1^] for CP204L gene. With these priors, the mean estimates of substitution rates for each gene were finally calculated by BEAST and ranged from 6.6×10^−4^ (CP204L) to 6.9×10^−4^ (B646L) subst/site/year (Table 3). These results are robust in terms of clock model, rate distribution, and population size parameters. The dated trees generated four lineages as previously described (Figure 6) and, again, the same isolates were found within these lineages. The four lineages were organized differently for the two genes: for B646L CP204L gene L1 and L2 were on the one hand and L3 and L4 on the other hand and in contrast, CP204L gene tree rendered different connections: L1, L2, and L4 together and L3 on the other hand. In both cases, the oldest lineage (TMRCA = 111 years) was L4, which gathers isolates from eastern Africa, the presumed birthplace of ASFV. It was followed by L1 (104 years), L2 (74 years), and L3 (47 years).

**Table 3:** Summary results of all tests done in BEAST for molecular clocking, models, evolution rates, and the TMRCA obtained.

gene	clock model	population size	evolutionary model	LRT	mean rate subst/site/year	TMRCA
					[95% HPD]	[95% HPD]
B646L	strict	constant	HKY+Г_5_	−1993.8	1.131×10^−4^	1622
					[7.6×10^−5^–1.5×10^−4^]	[1466–1768]
	UCLN	constant	HKY+Г_5_	−1593.4	6.9×10^−4^	1712
					[5.3×10^−4^–9.13×10^−4^]	[1465–1894]
CP204L	strict	constant	HKY+Г_5_	−2453.07	6.76×10^−5^	850
					[1.9×10^−5^–1.2×10^−4^]	[−204–1610]
	UCLN	constant	HKY+Г_5_	−2398.03	6.6×10^−4^	1700
					[5.57×10^−4^–8.75×10^−4^]	[1422–1891]

HPD = higher posterior density.

## Discussion

Because the localization of the major capsid protein VP72 in the virus core prevents exposure to circulating neutralizing antibodies, the corresponding B646L gene is not expected to be submitted to immune system pressure. Accordingly, only two amino-acid positions were detected as being under positive selection, suggesting no real impact on the evolutionary force. Therefore, the rate of substitutions of VP72 probably bears the information needed to estimate natural virus evolution. The VP72 homologues of closely related virus families have already been used in evolutionary studies [53] and for a decade in ASFV phylogenetic reconstructions. In contrast, P54 is an envelope protein and the pressure of the immune system on E183L evolution is revealed by nine amino-acid positions placed under positive selection, a strong non-synonymous bias and recombination events within the gene sequence. P32 is also an envelope protein but is involved in translation of viral genes by its interactions with hnRNP cellular protein [54]. In this context, mutations may be detrimental and, thus, the gene may be submitted to purifying selection, as corroborated by the detection of only three amino-acid positions under positive selection.

The evolution of ASFV mapped through partial genomic sequences and phylogenetic reconstructions shows a certain degree of complexity that may not be well represented by bifurcative methods. However, both bifurcative and network analyses in this study clearly provided clear clusterization into four major lineages (L1 to L4) while only three have been described so far [18]. Within these lineages, molecular signatures of the twenty-two already described genotypes were established and two new sub-lineages can be proposed, that is, Cro3.5 isolate, and TAN/08/MAZIMBU previously ascribed to genotype XV. Molecular signatures do not rely on the same number of substitutions and do not have an equal weight. For instance, L1 is characterized by 2 specific nucleic acid positions and 4 synonymous substitutions while L4 is defined by 12 sites and 13 nucleotide substitutions of which 3 are not synonymous. Within the L1 lineage, genotype I, which is the most represented in terms of sequences (Europe, West Africa, Caribbean and South America), is characterized by only one synonymous substitution (A216). This mutation, however, leads to an increase in ASFV codon preference for alanine (GCG to GCA) (http://www.kazusa.or.jp/codon/), which has surely helped to fix the substitution in the lineage for almost 60 years in three continents. Besides the molecular signature, the distance matrix also supports our proposal for new ASFV classification, which includes the previous genotype subdivision and additional sub-clustering.

ASFV shows a high evolutionary rate relative to that of other DNA viruses [55]. Consequently, this high substitution rate led to very recent TMRCAs: the most common ancestor of ASFV strains currently circulating emerged in around three centuries, in 1700. It is commonly agreed that ASFV is native of East Africa as the disease was first described in Kenya in 1921 after a first outbreak in 1903. Then, during decades ASFV showed a great ability to spread worldwide following major trade routes. In the wild, the virus is thought to be originally a virus of tick [11] as it infects argasids ticks of the *Ornithodoros* genus. *Ornithodoros,* which infest warthogs’ burrows, are endophile ticks, meaning that they need regular temperature and hygrometry. They also are photophobic so they do not spread out over long distances. ASFV is transmitted horizontally and vertically between ticks [56], [57] and between ticks and juvenile wild swine that stay in and close to their burrows. Under such circumstances, the virus is not supposed to spread much and its genetic drift over long periods may have resulted in isolated spots of diversity maintained by the sylvatic cycle with only few entries of new strains. In contrast, the domestic pig cycle is short with a dead-end disease essentially transmitted by contacts with pigs or pig meat and rarely by tick bites. Accordingly, the phylogenetic trees constructed in this study showed higher diversity within lineages of eastern and southern African isolates submitted to a sylvatic cycle than in lineages of domestic pigs from other regions. New variants are not easy to characterize because of the lack of sequence data from their parent lineage. For example, TAN/08/Mazimbu isolate collected in Tanzania in 2008 and originally placed in genotype XV [52] constitutes in this study a sub-lineage of L3. Thus, it should not be considered as a re-emergence of the TAN/01/1 isolate collected during an outbreak in Tanzania in 2001. Sixteen Italian isolates showed recombination events in the E183L gene and were subsequently removed from the corresponding reconstruction. This does not change the affiliation of these isolates to L1, as demonstrated by B646L and CP204L reconstructions (not shown). However, since all these isolates are linked together and show the same recombination events, assuming they all have emerged from a common recombined ancestor, the possibility that they will form a new sub-lineage within L1 has to be considered.

Two different genes and two methods were used to consolidate TMRCA estimation. Maximum likelihood method using PAML package showed that a strict molecular clock could not be validated for our set of genes. However, it did not provide consistent results when using a relaxed clock, with TMRCAs from −12000 to 1500. In contrast, the Bayesian approach generated consistent results, B646L and CP204L analyses dating a TMRCA around 1700 AD with a rate of subst/site/year estimated to be around 6.7×10^−4^. As illustrated by the E183L gene analysis, the role of the immune system on sequence variability may influence the sequence evolution of some ASFV genes which may consequently render a biased TMRCA (data not shown for the E183L gene in this paper). Therefore, the natural evolution of the virus may be well represented by B646L and CP204L genes in which neither recombination events nor non-synonymous bias or too many codons under positive selection were detected. The TMRCA scale going back to 1700 AD for all ASFV isolates can be considered with confidence since within this scale, the TMRCA of lineage L1-1 and L1-2 were 1943/1955 (for B646L/CP204L genes) and 1990 (for both genes), respectively. L1-1 is supposed to have emerged in the late 1950s [16] and L1-2 includes mainly isolates from Madagascar that were first introduced in 1998 [58]. The substitution rates determined in this study were much higher than expected relative to other large dsDNA viruses like gamma-herpes viruses of vertebrate (10^−9^ subs/site/year) or even small dsDNA viruses like the John Cunningham polyomavirus (10^−7^ subs/site/year) [55]. With a substitution rate between 10^−4^ and 10^−5^, ASFV approaches RNA viruses that usually have 10^−2^ to 10^−5^ subs/site/year [59].

Like many other large dsDNA viruses [60], ASFV may have co-evolved with its host. This means a long and ancient history of the virus in the wild. A high substitution rate combined with recent TMRCAs is not consistent with ancient co-evolution of viruses and their hosts, which in contrast should lead to a low rate of substitution [61]. However, for a virus that replicates at a high level in its host, a low rate of subst/site/replication can still lead to an increased accumulation of diversity, which in turns generates high rates of subst/site/year [62]. This has been described for highly contagious viruses that induce acute forms of infection and show a higher observed rate of subst/site/year [63]. In contrast, an asymptomatic infection of the host may not allow an exponential replication rate. ASFV presents these two characteristics, being asymptomatic in natural African wild swine and soft ticks and highly contagious and lethal in domestic pigs. Consequently, a stochastic event may have occurred around 300 years from now that would explain the emergence of an ancestor common to all known ASFV isolated so far in domestic and wild pigs. Our assumption is based on the introduction of domestic pigs in Africa. Domestic pigs have Eurasian and North African ancestral wild boar origins [64]. Even though Plug (2001) [65], claimed pigs were introduced in South Africa between the 3^rd^ and 7^th^ centuries, Swart (2010) [66] believes domestic pigs were not present in eastern and southern African livestock because of the nomadic lifestyle of pastoralists at this time. Domestic pigs may have been brought first by the Chinese around 600 years ago [67] then by the Portuguese 300 to 400 years ago [68], both during their exploration and conquest period for trade opportunities. The assumption of pig introduction from Europe and the Far East was confirmed by phylogenetic analysis revealing contributions of both origins in the genetic pattern of local African pigs [69]. Following the circumnavigation of Africa by European nations during 15^th^ - 17^th^ centuries, pig breed types were introduced during 16^th^ and 17^th^ centuries [66], mainly by the Portuguese to the East Africa coast via Goa. Pig breeding diffused then slowly northward from Mozambique [68]. The Portuguese did not colonize Kenya for settlement but as a step to India and definitely left the country in 1720 after being defeated by the Arabs in 1698. Despite Arab colonization and the pig-eating taboo, domestic pigs were eaten by ethnic groups like the Waata in southern Kenya since the 16^th^ century and called Walyankuru: “those who eat pig” [70]. This may have enabled the virus to spread silently among sensitive pig species. Kenya was then colonized by the British. At the end of the 19^th^ century, the extensive pig industry in the native region of ASFV started after a massive loss of bovine cattle due to rinderpest outbreak. Pigs were massively imported for breeding by colonizers from Seychelles in 1904 and from England in 1905. Pig farming was free ranging at this time and the first outbreak of ASF was reported in 1907. Trade routes and virus resistance in the environment then enabled further spreading of ASFV.

## Supporting Information

Table S1 **List of ASFV and NCLDVs isolates and corresponding genes used in this study.**
(DOCX)

File S1 **Alignment of NCLDVs capsid proteins.**
(FAS)
