# Supplementary material for: Comprehensive Phylogenetic Reconstructions of African Swine Fever Virus: Proposal for a New Classification and Molecular Dating of the Virus
Source: PLoS One. 2013 Jul 25;8(7):e69662. doi: 10.1371/journal.pone.0069662 (PMC3723844; doi:10.1371/journal.pone.0069662)
Supplement: Table S1 — List of ASFV and NCLDVs isolates and corresponding genes used in this study. (DOCX) [file pone.0069662.s001.docx]

Supporting information: ASFV isolates and corresponding genes used in this study. Lineages and corresponding genotypes are indicated.

| **Isolate** | **Country** | **Year** | **Host species** | **GenBank accession number** | | | **Genotype** | **Lineage** | **Reference** |
| --- | --- | --- | --- | --- | --- | --- | --- | --- | --- |
|  |  |  |  | **B646L/MCP** | **E183L** | **CP204L** |  |  |  |
| 608 | NC | NC | NC | - | - | AF462274 | - | L1 | Hernaez *et al*., 2001 |
| 646 | Spain | 1969 | Domestic pig | FJ174351 | FJ174392 | - | I | L1.1 | Gallardo *et al.* 2009 |
| 1207 | NC | NC |  | - | - | AF462273 | - | L1 | Hernaez *et al*., 2001 |
| 24823 | RSA | 1975 | NC | DQ250110 | - | - | XX | L2.2.4 | Boshoff *et al.* 2007 |
| 04/Ol/02 | Italy | 2002 | Domestic pig | - | FR681815 | - | I | L1.1 | Giammarioli *et al.* 2011 |
| 1/Nu/97 | Italy | 1997 | Domestic pig | FR668398 | FR681812 | - | I | L1.1 | Giammarioli *et al.* 2011 |
| 11/Og/04 | Italy | 2004 | Domestic pig | FR668403 | FR681817 | - | I | L1.1 | Giammarioli *et al.* 2011 |
| 13/Nu/04 | Italy | 2004 | Domestic pig | - | FR681818 | - | I | L1.1 | Giammarioli *et al.* 2011 |
| 16/Og/04 | Italy | 2004 | Domestic pig | FR682502 | FR681819 | - | I | L1.1 | Giammarioli *et al.* 2011 |
| 18/Nu/04 | Italy | 2004 | Domestic pig | FR677326 | FR681820 | - | I | L1.1 | Giammarioli *et al.* 2011 |
| 2/Og/97 | Italy | 1997 | Domestic pig | FR668399 | FR681813 | - | I | L1.1 | Giammarioli *et al.* 2011 |
| 22/Nu/04 | Italy | 2004 | Domestic pig | FR668405 | FR681821 | - | I | L1.1 | Giammarioli *et al.* 2011 |
| 23/Or/04 | Italy | 2004 | Domestic pig | FR668406 | FR681822 | - | I | L1.1.5 | Giammarioli *et al.* 2011 |
| 24/Or/04 | Italy | 2004 | Domestic pig | FR668407 | FR681823 | - | I | L1.1.5 | Giammarioli *et al.* 2011 |
| 25/Nu/04 | Italy | 2004 | Domestic pig | FR668408 | FR681824 | - | I | L1.1.5 | Giammarioli *et al.* 2011 |
| 26/Ss/04 | Italy | 2004 | Domestic pig | - | FR681825 | - | I | L1.1 | Giammarioli *et al.* 2011 |
| 3/Og/98 | Italy | 1998 | Domestic pig | FR668400 | FR681814 | - | I | L1.1 | Giammarioli *et al.* 2011 |
| 30/Ol/04 | Italy | 2004 | Domestic pig | FR668410 | FR681826 | - | I | L1.1.3 | Giammarioli *et al.* 2011 |
| 36/Ss/05 | Italy | 2005 | Domestic pig | FR668411 | FR681827 | - | I | L1.1 | Giammarioli *et al.* 2011 |
| 38/Ss/07 | Italy | 2007 | Domestic pig | FR668412 | FR681828 | - | I | L1.1 | Giammarioli *et al.* 2011 |
| 41/Og/07 | Italy | 2007 | Wild boar | FR668413 | FR681829 | - | I | L1.1 | Giammarioli *et al.* 2011 |
| 42/Og/07 | Italy | 2007 | Wild boar | FR668414 | FR681830 | - | I | L1.1 | Giammarioli *et al.* 2011 |
| 43/Og/07 | Italy | 2007 | Wild boar | FR668415 | FR681831 | - | I | L1.1 | Giammarioli *et al.* 2011 |
| 46/Ca/08 | Italy | 2008 | Domestic pig | - | FR681832 | - | I | L1.1 | Giammarioli *et al.* 2011 |
| 47/Ss/08 | Italy | 2008 | Domestic pig | - | FR681833 | - | I | L1.1 | Giammarioli *et al.* 2011 |
| 48/Ss/08 | Italy | 2008 | Domestic pig | - | FR681834 | - | I | L1.1 | Giammarioli *et al.* 2011 |
| 5/Ca/02 | Italy | 2002 | Domestic pig | FR668402 | FR681816 | - | I | L1.1.3 | Giammarioli *et al.* 2011 |
| 51/Nu/09 | Italy | 2009 | Domestic pig | FR668419 | FR681835 | - | I | L1.1 | Giammarioli *et al.* 2011 |
| 52/Nu/09 | Italy | 2009 | Domestic pig | - | FR681836 | - | I | L1.1 | Giammarioli *et al.* 2011 |
| 53/Nu/09 | Italy | 2009 | Domestic pig | FR677327 | FR681837 | - | I | L1.1 | Giammarioli *et al.* 2011 |
| 9PHYC_1 | - | - | - | Q8QNG3 | - | - | - | - | pfam04451 |
| 9PHYC_2 | - | - | *Feldmannia irregularis* | Q6XLY6 | - | - | - | - | pfam04451 |
| 9PHYC_3 | - | - | - | Q4A348 | - | - | - | - | pfam04451 |
| 9VIRU_1 | - | - | Korean fish | Q6QNG8 | - | - | - | - | pfam04451 |
| 9VIRU_2 | - | - | - | Q5GAH7 | - | - | - | - | pfam04451 |
| 9VIRU_3 | - | - | *Diadromus pulchellus* | Q8JKF0 | - | - | - | - | pfam04451 |
| 9VIRU_4 | - | - | *-* | Q4H484 | - | - | - | - | pfam04451 |
| 9VIRU_5 | - | - | *-* | Q9QAJ0 | - | - | - | - | pfam04451 |
| 9VIRU_6 | China | - | *-* | Q6JLA3 | - | - | - | - | pfam04451 |
| 98/ASF/NG | Nigeria | 1998 | Domestic pig | AF159503 | - | - | I | L1.1 | Bastos *et al.* 2003 |
| Ali61 | Spain | 1961 | Domestic pig | FJ154445 | FJ174384 | - | I | L1.1 | Gallardo *et al.* 2009 |
| Almodovar99 | Portugal | 1999 | Domestic pig | DQ028306 | DQ028315 | - | I | L1.1 | Duarte *et al.* 2005 |
| Almodovar99E2 | Portugal | 1999 | *Ornithodoros* tick | DQ028308 | DQ028316 | - | I | L1.1 | Duarte *et al.* 2005 |
| Almodovar99NE1 | Portugal | 1999 | *Ornithodoros* tick | DQ028309 | DQ028317 | - | I | L1.1 | Duarte *et al.* 2005 |
| Ambaton01 | Madagascar | 2001 | Domestic pig | KC610529 | - | - | II | L1.2 | This study |
| Ambilo03 | Madagascar | 2003 | Domestic pig | KC610528 | KC610539 | KC610540 | II | L1.2 | This study |
| Ambovo99 | Madagascar | 1999 | Domestic pig | KC610527 | - | - | II | L1.2 | This study |
| Ampani99 | Madagascar | 1999 | Domestic pig | KC610526 | EU620685 | EU620686 | II | L1.2 | This study |
| ANG/70 | Angola | 1970 | Domestic pig | AF301542 | EU874327 | EU874271 | I | L1.1 | Bastos *et al.* 2003 |
| Ang72 | Angola | 1972 | Domestic pig | FJ174378 | FJ174424 | - | I | L1.1 | Gallardo *et al.* 2009 |
| Antana00 | Madagascar | 2000 | Domestic pig | KC610525 | - | - | II | L1.2 | This study |
| Antani03 | Madagascar | 2003 | Domestic pig | KC610523 | EU620681 | EU620687 | II | L1.2 | This study |
| Antsir99 | Madagascar | 1999 | Domestic pig | - | KC610538 | KC610541 | II | L1.2 | This study |
| Antsira02 | Madagascar | 2002 | Domestic pig | - | - | KC610542 | II | L1.2 | This study |
| AR158_C675L | - | - | Chlorella | YP_001498756 | - | - | - | - | pfam04451 |
| Arivo01 | Madagascar | 2001 | Domestic pig | - | - | KC610543 | II | L1.2 | This study |
| ATCV1_Z280L | Germany | - | *Chlorella sp*. SAG 3.83 | YP_001426761 | - | - | - | - | pfam04451 |
| ATCV1_Z151L | Germany | - | *Chlorella sp*. SAG 3.83 | YP_001426632 | - | - | - | - | pfam04451 |
| ATCV1_Z506L | Germany | - | *Chlorella sp*. SAG 3.83 | YP_001426987 | - | - | - | - | pfam04451 |
| ATCV1_Z558L | Germany | - | *Chlorella sp*. SAG 3.83 | YP_001427039 | - | - | - | - | pfam04451 |
| ATCV1_Z664R | Germany | - | *Chlorella sp*. SAG 3.83 | YP_001427145 | - | - | - | - | pfam04451 |
| Av71 | Spain | 1971 | Domestic pig | FJ174349 | FJ174391 | - | I | L1.1 | Gallardo *et al.* 2009 |
| Avara02 | Madagascar | 2002 | Domestic pig | KC610522 | KC610537 | KC610544 | II | L1.2 | This study |
| Awoshie99 | Ghana | 1999 | NC | AF504885 | - | - | I | L1.1 | Bastos *et al.* 2003 |
| B74 | Spain | 1974 | Domestic pig | FJ174350 | FJ174393 | - | I | L1.1 | Gallardo *et al.* 2009 |
| Ba71V | Spain | 1971 |  | FJ174348 | FJ174390 | M96354 | I | L1.1 | Bastos *et al.* 2003 |
| BAN/91/1 | Malawi | 1991 | *Sus scrofa* | AY351501 | EU874348 | EU874260 | VIII | L3.1 | Lubisi *et al.* 2005 |
| Barrancos93 | Portugal | 1993 | Domestic pig | DQ028307 | DQ028318 | - | I | L1.1 | Duarte *et al.* 2005 |
| Bartlett2 | Kenya | 1959 | *Phaecochoerus aethiopicus* | AY351532 | - | - | X | L4.2.2.2.1 | Lubisi *et al.* 2005 |
| BEL85 | Belgium | 1985 | Domestic pig | AF449466 | - | - | I | L1.1 | Bastos *et al.* 2003 |
| BEN97/4 | Benin | 1997 | Domestic pig | AY972164 | - | - | I | L1.1 | Phologane *et al.* 2005 |
| Benin97/1 | Benin | 1997 | Domestic pig | AF302816 | AM712239 | AM712239 | I | L1.1 | Bastos *et al.* 2003 |
| Betrok99 | Madagascar | 1999 | Domestic pig | - | KC610536 | KC610556 | II | L1.2 | This study |
| Bongera/83 | Malawi | 1983 | Domestic pig | - | X84905 | - | VIII | L3.1 | Sun *et al.* 1995 |
| BOT/99/1 | Botswana | 1999 | Domestic pig | - | EU874382 | - | III | L2.2.1 | Heath *et al.* 2008 |
| Brazil78 | Brazil | 1978 | Domestic pig | FJ238537 | FJ238535 | - | I | L1.1 | Gallardo *et al.* 2009 |
| Brazil79 | Brazil | 1979 | NC | AF302809 | - | - | I | L1.1 | Bastos *et al.* 2003 |
| BUR/84/1 | Burundi | 1984 | Domestic pig | AF449463 | EU874364 | EU874298 | X | L4.2.2.2.3 | Bastos *et al.* 2003 |
| BUR/84/2 | Burundi | 1984 | Domestic pig | AF449464 | - | - | X | L4.2.2.2.3 | Bastos *et al.* 2003 |
| BUR/90/1 | Burundi | 1990 | Domestic pig | AF449472 | EU874363 | EU874299 | X | L4.2.2.2.3 | Bastos *et al.* 2003 |
| BUR/90/3 | Burundi | 1990 | *Sus scrofa* | AY351525 | - | - | X | L4.2.2.2.3 | Lubisi *et al.* 2005 |
| Ca04.1 | Italy | 2004 | Domestic pig | FR668270 | FR668271 | - | I | L1.1 | Giammarioli *et al.* 2011 |
| Ca78 | Italy | 1978 | Domestic pig | FJ174357 | FJ174401 | - | I | L1.1 | Nix *et al.* 2006 |
| Ca97 | Italy | 1997 | Domestic pig | FJ174371 | FJ174416 | - | I | L1.1 | Gallardo *et al.* 2009 |
| CAM/82 | Cameroun | 1982 | Domestic pig | AF301544 | - | - | I | L1.1 | Bastos *et al.* 2003 |
| CAM/02/1 | Cameroun | 2002 | NC | - | EU874323 | EU874267 | I | L1.1 | Heath *et al.* 2008 |
| CAM/02/2 | Cameroun | 2002 | NC | - | EU874324 | EU874268 | I | L1.1 | Heath *et al.* 2008 |
| CAM/02/3 | Cameroun | 2002 | NC | - | EU874325 | EU874269 | I | L1.1 | Heath *et al.* 2008 |
| CAM/02/4 | Cameroun | 2002 | NC | - | EU874326 | EU874270 | I | L1.1 | Heath *et al.* 2008 |
| CAM/85/4 | Cameroun | 1985 | NC | - | EU874322 | EU874272 | I | L1.1 | Bastos *et al.* 2003 |
| CHG/88/1 | Zambia | 1988 | *Sus scrofa* | AY351552 | - | - | VIII | L3.1 | Lubisi *et al.* 2005 |
| CHJ/89/1 | Zambia | 1989 | *Sus scrofa* | AY351519 | EU874346 | EU874262 | VIII | L3.1 | Lubisi *et al.* 2005 |
| CHK/89/2 | Zambia | 1989 | *Sus scrofa* | AY351526 | - | - | VIII | L3.1 | Lubisi *et al.* 2005 |
| CHM/88/1 | Zambia | 1988 | *Sus scrofa* | AY351520 | - | - | VIII | L3.1 | Lubisi *et al.* 2005 |
| Chrôme01 | Madagascar | 2001 | Domestic pig | KC610524 | EU620682 | EU620688 | II | L1.2 | This study |
| CM96 | Ivory Coast | 1996 | Domestic pig | - | - | - | I | L1.1 | This study |
| Co61 | Spain | 1961 | Domestic pig | FJ174346 | FJ174386 | - | I | L1.1 | Gallardo *et al.* 2009 |
| Co62 | Spain | 1962 | Domestic pig | FJ174347 | FJ174387 | - | I | L1.1 | Gallardo *et al.* 2009 |
| Co68 | Spain | 1968 | Domestic pig | FJ238538 | FJ174388 | - | I | L1.1 | Gallardo *et al.* 2009 |
| Coimbra87 | Portugal | 1987 | Domestic pig | DQ028310 | DQ028319 | - | I | L1.1 | Duarte *et al.* 2005 |
| Con09/Abo | RDC | 2009 | Domestic pig | - | HQ645951 | - | IX | L4.1 | Gallardo *et al.* 2011 |
| Con09/Bzz020 | RDC | 2009 | Domestic pig | - | HQ645950 | - | IX | L4.1 | Gallardo *et al.* 2011 |
| Con09/Ni16 | RDC | 2009 | Domestic pig | - | HQ645948 | - | IX | L4.1 | Gallardo *et al.* 2011 |
| Con09/Pk45 | RDC | 2009 | Domestic pig | - | HQ645952 | - | IX | L4.1 | Gallardo *et al.* 2011 |
| Con09/PN003 | RDC | 2009 | Domestic pig | - | HQ645949 | - | IX | L4.1 | Gallardo *et al.* 2011 |
| Cro3.5 | RSA | 1996 | *Ornithodoros* tick | AY578691 | - | - | NA | L2.3.4 | Zsak *et al.* 2005 |
| Cro1.2 | RSA | 1996 | *Ornithodoros* tick | AY578690 | - | - | XX | L2.2.4 | Zsak *et al.* 2005 |
| CV97 | Cape Verde | 1997 | Domestic pig | FJ174380 | FJ174427 | - | I | L1.1 | Gallardo *et al.* 2009 |
| CV98 | Cape Verde | 1998 | Domestic pig | FJ174381 | FJ174428 | - | I | L1.1 | Gallardo *et al.* 2009 |
| CVR/Tet20 | Nigeria | 2005 | NC | GQ427180 | - | - | I | L1.1 | Owolodun *et al.* 2010 |
| CVR/Tet21 | Nigeria | 2005 | NC | GQ427181 | - | - | I | L1.1 | Owolodun *et al.* 2010 |
| CVR/Tet29 | Nigeria | 2006 | NC | GQ427183 | - | - | I | L1.1 | Owolodun *et al.* 2010 |
| Dakar59 | Sénégal | 1959 |  | AF301538 | - | - | I | L1.1 | Bastos *et al.* 2003 |
| Davis | Kenya | 1959 | *Phaecochoerus aethiopicus* | AY351527 | - | - | X | L4.2.2.2.3 | Lubisi *et al.* 2005 |
| DED/89/1 | Malawi | 1989 | *Sus scrofa* | AY351502 | EU874349 | EU874256 | VIII | L3.1 | Lubisi *et al.* 2005 |
| DED/91/1 | Malawi | 1991 | *Sus scrofa* | AY351503 | - | - | VIII | L3.1 | Lubisi *et al.* 2005 |
| Dedza | Malawi | 1986 | Domestic pig | AF449479 | - | - | VIII | L3.1 | Bastos *et al.* 2003 |
| Doig | Kenya | 1957 | *Phaecochoerus aethiopicus* | AY351528 | - | - | X | L4.2.2.2.2 | Lubisi *et al.* 2005 |
| DomRep79 | Dominican Republic | 1978 | NC | AF302810 | FJ238534 | - | I | L1.1 | Bastos *et al.* 2003 |
| DOWA | Malawi | 1986 | *Sus scrofa* | AY351509 | EU874350 | EU874261 | VIII | L3.1 | Lubisi *et al.* 2005 |
| DR2 | Dominican Republic | 1979 | Domestic pig | ASVMCPC | - | - | I | L1.1 | Yu *et al.* 1996 |
| DR78 | Dominican Republic | 1978 | Domestic pig | - | - | - | I | L1.1 | This study |
| E70 | Spain | 1970 | Domestic pig | AY578692 | FJ174389 | AF462272 | I | L1.1 | Zsak *et al.* 2005 |
| E75 | Spain | 1975 | Domestic pig | AY578693 | FJ174394 | AF462271 | I | L1.1 | Zsak *et al.* 2005 |
| F6 | RSA | 1996 | *Ornithodoros* tick | AY578694 | - | - | XIX | L2.2.3 | Zsak *et al.* 2005 |
| Faharet98 | Madagascar | 1998 | Domestic pig | - | KC610535 | KC610545 | II | L1.2 | This study |
| Fandria01 | Madagascar | 2001 | Domestic pig | - | - | KC610546 | II | L1.2 | This study |
| Fianara00 | Madagascar | 2000 | Domestic pig | KC610521 | KC610534 | KC610547 | II | L1.2 | This study |
| FRG3G | - | - | *Salientia* | Q67473 | - | - | - | - | pfam04451 |
| FR483_N254L | France | - | *Chlorella sp.* strain Pbi | YP_001425886 | - | - | - | - | pfam04451 |
| FR483_N470L | France | - | *Chlorella sp.* strain Pbi | YP_001426102 | - | - | - | - | pfam04451 |
| Fr64 | France | 1964 | Domestic pig | FJ174374 | FJ174421 | - | I | L1.1 | Nix *et al.* 2006 |
| GAM/1/00 | Gambia | 2000 | Domestic pig | AF449478 | - | - | I | L1.1 | Bastos *et al.* 2003 |
| Gara08 | Madagascar | 2008 | Domestic pig | - | - | KC610548 | II | L2.1 | This study |
| Gasson | Kenya | 1961 | *Sus scrofa* | AY351529 | - | - | X | L4.2.2.2.1 | Lubisi *et al.* 2005 |
| Georgia2007 | Georgia | 2007 | Domestic pig | AM999764 | AM999765 | AM999766 | II | L1.2 | Rowland *et al.* 2008 |
| GHA/1/00 | Ghana | 2000 | Domestic pig | AF504888 | - | - | I | L1.1 | Bastos *et al.* 2003 |
| Ghana | Ghana | 2000 | NC | AF504889 | - | - | I | L1.1 | Bastos *et al.* 2003 |
| GHANA/02/1 | Ghana | 2002 | NC | - | EU874328 | EU874293 | I | L1.1 | Heath *et al.* 2008 |
| GR21/11 | RSA | 1978 | *Ornithodoros* tick | FJ455836 | - | - | XX | L2.2.4 | Arnot *et al.* 2009 |
| GR21/23 | RSA | 1978 | *Ornithodoros* tick | FJ455837 | - | - | XX | L2.2.4 | Arnot *et al.* 2009 |
| GR22/6 | RSA | 1978 | *Ornithodoros* tick | FJ455838 | - | - | XX | L2.2.4 | Arnot *et al.* 2009 |
| GR44A2 | RSA | 1979 | *Ornithodoros* tick | FJ455835 | - | - | XX | L2.3.2 | Arnot *et al.* 2009 |
| GUL/88/1 | Zambia | 1988 | *Sus scrofa* | AY351521 | - | - | VIII | L3.1 | Lubisi *et al.* 2005 |
| Hai81 | Haïti | 1981 | Domestic pig | FJ174375 | FJ238536 | - | I | L1.1 | Gallardo *et al.* 2009 |
| HindeI | Kenya | 1954 | Suids | AY351530 | - | - | X | L4.2.2.2.2 | Lubisi *et al.* 2005 |
| HindeII | Kenya | 1959 | Domestic pig | AF449480 | - | - | X | L4.2.2.2.2 | Bastos *et al.* 2003 |
| HOL86 | Netherland | 1986 | Domestic pig | AF449467 | - | - | I | L1.1 | Bastos *et al.* 2003 |
| ht79 | Haïti | 1979 | Domestic pig | AY578695 | - | - | I | L1.1 | Zsak *et al.* 2005 |
| Hu90 | Spain | 1990 | Domestic pig | FJ174355 | FJ174399 | - | I | L1.1 | Gallardo *et al.* 2009 |
| Hu94 | Spain | 1994 | Domestic pig | FJ174356 | FJ174400 | - | I | L1.1 | Gallardo *et al.* 2009 |
| IC/3/96 | Ivory Coast | 1996 | Domestic pig | AF504882 | - | - | I | L1.1 | Bastos *et al.* 2003 |
| IC/5/76 | Ivory Coast | 1996 | NC | AF504883 | - | - | I | L1.1 | Bastos *et al.* 2003 |
| IC/1/96 | Ivory Coast | 1996 | Domestic pig | AF302814 | - | - | I | L1.1 | Bastos *et al.* 2003 |
| IC/2/96 | Ivory Coast | 1996 | Domestic pig | AF302815 | EU874319 | EU874282 | I | L1.1 | Bastos *et al.* 2003 |
| IC96 | Ivory Coast | 1996 | Domestic pig | FJ174379 | FJ174429 | - | I | L1.1 | Gallardo *et al.* 2009 |
| IIV3 | - | - | Invertebrate | Q197E6 | - | - | - | - | pfam04451 |
| IIV6 |  |  | Invertebrate | Q05815 | - | - | - | - | pfam04451 |
| IRV1 | - | - | *Tipula* | P18162 | - | - | - | - | pfam04451 |
| JON/89/13 | Zambia | 1989 | Domestic pig | AF449469 | - | - | VIII | L3.1 | Bastos *et al.* 2003 |
| K1 | RSA | 1996 | *Ornithodoros* tick | AY578696 | - | - | III | L2.2.1 | Zsak *et al.* 2005 |
| KAB/62 | Zambia | 1983 | *Ornithodoros* tick | AY351522 | EU874331 | EU874289 | XI | L3.2 | Lubisi *et al.* 2005 |
| KAB/94/1 | Kenya | 1994 | Domestic pig | AY972163 | - | - | X | L4.2.2.2.3 | Phologane *et al.* 2005 |
| KAC/91/2 | Malawi | 1991 | *Sus scrofa* | AY351504 | - | - | VIII | L3.1 | Lubisi *et al.* 2005 |
| KAL/88/1 | Zambia | 1988 | Domestic pig | AF449468 | - | - | VIII | L3.1 | Bastos *et al.* 2003 |
| KANA/89/1 | Zambia | 1989 | *Sus scrofa* | AY351523 | - | - | VIII | L3.1 | Lubisi *et al.* 2005 |
| Kat67 | RDC | 1967 | Domestic pig | FJ174377 | FJ174423 | - | I | L1.1 | Gallardo *et al.* 2009 |
| Katanga63 | RDC | 1963 | Domestic pig | AF301540 | - | - | I | L1.1 | Bastos *et al.* 2003 |
| KAV/89/1 | Zambia | 1989 | *Ornithodoros* tick | AF449470 | - | - | VIII | L3.1 | Bastos *et al.* 2003 |
| KEN/05/1 | Kenya | 2005 | NC | - | EU874368 | EU874301 | IX | L4.1 | Heath *et al.* 2008 |
| Ken05.DPk16 | Kenya | 2005 | Domestic pig | HM745264 | HM745347 | HM745369 | X | L4.2.2.1 | Gallardo *et al.* 2011 |
| Ken05.DPk18 | Kenya | 2005 | Domestic pig | HM745265 | HM745348 | HM745370 | X | L4.2.2.1 | Gallardo *et al.* 2011 |
| Ken05.DPk2 | Kenya | 2005 | Domestic pig | HM745263 | HM745346 | HM745368 | X | L4.2.2.1 | Gallardo *et al.* 2011 |
| Ken05.DPk21 | Kenya | 2005 | Domestic pig | HM745266 | HM745349 | HM745371 | X | L4.2.2.1 | Gallardo *et al.* 2011 |
| Ken05.DPk27 | Kenya | 2005 | Domestic pig | HM745267 | HM745350 | HM745372 | X | L4.2.2.1 | Gallardo *et al.* 2011 |
| Ken05.DPN15 | Kenya | 2005 | Domestic pig | HM745269 | HM745352 | HM745374 | X | L4.2.2.1 | Gallardo *et al.* 2011 |
| Ken05.DPN2 | Kenya | 2005 | Domestic pig | HM745268 | HM745351 | HM745373 | X | L4.2.2.1 | Gallardo *et al.* 2011 |
| Ken05.DPN23 | Kenya | 2005 | Domestic pig | HM745270 | HM745353 | HM745375 | X | L4.2.2.1 | Gallardo *et al.* 2011 |
| Ken05.DPU1 | Kenya | 2005 | Domestic pig | HM745271 | HM745354 | HM745376 | X | L4.2.2.1 | Gallardo *et al.* 2011 |
| Ken05.DPU11 | Kenya | 2005 | Domestic pig | HM745273 | HM745356 | HM745378 | X | L4.2.2.1 | Gallardo *et al.* 2011 |
| Ken05.DPU2 | Kenya | 2005 | Domestic pig | HM745272 | HM745355 | HM745377 | X | L4.2.2.1 | Gallardo *et al.* 2011 |
| Ken05.DPU22 | Kenya | 2005 | Domestic pig | HM745274 | HM745357 | HM745379 | X | L4.2.2.1 | Gallardo *et al.* 2011 |
| Ken05/Tk1 | Kenya | 2005 | *Ornithodoros* tick | HM745253 | HM745336 | HM745358 | X | L4.2.1 | Gallardo *et al.* 2011 |
| Ken05/Tk10 | Kenya | 2005 | *Ornithodoros* tick | HM745262 | HM745345 | HM745367 | X | L4.2.2.1 | Gallardo *et al.* 2011 |
| Ken05/Tk2 | Kenya | 2005 | *Ornithodoros* tick | HM745254 | HM745337 | HM745359 | X | L4.2.1 | Gallardo *et al.* 2011 |
| Ken05/Tk3 | Kenya | 2005 | *Ornithodoros* tick | HM745255 | HM745338 | HM745360 | X | L4.2.1 | Gallardo *et al.* 2011 |
| Ken05/Tk4 | Kenya | 2005 | *Ornithodoros* tick | HM745256 | HM745339 | HM745361 | X | L4.2.1 | Gallardo *et al.* 2011 |
| Ken05/Tk5 | Kenya | 2005 | *Ornithodoros* tick | HM745257 | HM745340 | HM745362 | X | L4.2.2.1 | Gallardo *et al.* 2011 |
| Ken05/Tk6 | Kenya | 2005 | *Ornithodoros* tick | HM745258 | HM745341 | HM745363 | X | L4.2.1 | Gallardo *et al.* 2011 |
| Ken05/Tk7 | Kenya | 2005 | *Ornithodoros* tick | HM745259 | HM745342 | HM745364 | X | L4.2.2.1 | Gallardo *et al.* 2011 |
| Ken05/Tk8 | Kenya | 2005 | *Ornithodoros* tick | HM745260 | HM745343 | HM745365 | X | L4.2.1 | Gallardo *et al.* 2011 |
| Ken05/Tk9 | Kenya | 2005 | *Ornithodoros* tick | HM745261 | HM745344 | HM745366 | X | L4.2.2.1 | Gallardo *et al.* 2011 |
| Ken06.B1 | Kenya | 2006 | Domestic pig | FJ154434 | FJ174441 | - | IX | L4.1 | Gallardo *et al.* 2009 |
| Ken06.B2 | Kenya | 2006 | Domestic pig | FJ154435 | FJ174442 | - | IX | L4.1 | Gallardo *et al.* 2009 |
| Ken06.B3 | Kenya | 2006 | Domestic pig | FJ154436 | FJ174443 | - | IX | L4.1 | Gallardo *et al.* 2009 |
| Ken06.B4 | Kenya | 2006 | Domestic pig | FJ154437 | FJ174444 | - | IX | L4.1 | Gallardo *et al.* 2009 |
| Ken06.B5 | Kenya | 2006 | Domestic pig | FJ154438 | FJ174445 | - | IX | L4.1 | Gallardo *et al.* 2009 |
| Ken06.Bus | Kenya | 2006 | Domestic pig | FJ154439 | FJ174446 | - | IX | L4.1 | Gallardo *et al.* 2009 |
| Ken06.Kis | Kenya | 2006 | Domestic pig | FJ154440 | FJ174447 | - | IX | L4.1 | Gallardo *et al.* 2009 |
| Ken07.Eld1 | Kenya | 2007 | Domestic pig | FJ154441 | FJ174438 | - | IX | L4.1 | Gallardo *et al.* 2009 |
| Ken07.Eld2 | Kenya | 2007 | Domestic pig | FJ154442 | FJ174439 | - | IX | L4.1 | Gallardo *et al.* 2009 |
| Ken07.Kia | Kenya | 2007 | Domestic pig | FJ154443 | FJ174437 | - | IX | L4.1 | Gallardo *et al.* 2009 |
| Ken07.Nak | Kenya | 2007 | Domestic pig | FJ154444 | FJ174440 | - | IX | L4.1 | Gallardo *et al.* 2009 |
| Ken08Tk.2/1 | Kenya | 2008 | *Ornithodoros* tick | HM745275 | HM745323 | HM745380 | X | L4.2.2.1 | Gallardo *et al.* 2011 |
| Ken08Tk.2/3 | Kenya | 2008 | *Ornithodoros* tick | HM745276 | HM745324 | HM745381 | X | L4.2.2.1 | Gallardo *et al.* 2011 |
| Ken08WH/4 | Kenya | 2008 | *Phacochoerus africanus* | HM745285 | HM745333 | HM745390 | IX | L4.1 | Gallardo *et al.* 2011 |
| Ken08WH/5 | Kenya | 2008 | *Phacochoerus africanus* | HM745286 | HM745334 | HM745392 | IX | L4.1 | Gallardo *et al.* 2011 |
| Ken08WH/8 | Kenya | 2008 | *Phacochoerus africanus* | HM745287 | HM745335 | HM745391 | IX | L4.1 | Gallardo *et al.* 2011 |
| Ken09Tk.13/1 | Kenya | 2009 | *Ornithodoros* tick | HM745277 | HM745325 | HM745382 | X | L4.2.2.1 | Gallardo *et al.* 2011 |
| Ken09Tk.13/2 | Kenya | 2009 | *Ornithodoros* tick | HM745278 | HM745326 | HM745383 | X | L4.2.2.1 | Gallardo *et al.* 2011 |
| Ken09Tk.15/4 | Kenya | 2009 | *Ornithodoros* tick | HM745279 | HM745327 | HM745384 | X | L4.2.2.1 | Gallardo *et al.* 2011 |
| Ken09Tk.15/6 | Kenya | 2009 | *Ornithodoros* tick | HM745280 | HM745328 | HM745385 | X | L4.2.2.1 | Gallardo *et al.* 2011 |
| Ken09Tk.19/11 | Kenya | 2009 | *Ornithodoros* tick | HM745283 | HM745331 | HM745388 | X | L4.2.2.1 | Gallardo *et al.* 2011 |
| Ken09Tk.19/2 | Kenya | 2009 | *Ornithodoros* tick | HM745281 | HM745329 | HM745386 | X | L4.2.2.1 | Gallardo *et al.* 2011 |
| Ken09Tk.19/7 | Kenya | 2009 | *Ornithodoros* tick | HM745282 | HM745330 | HM745387 | X | L4.2.2.1 | Gallardo *et al.* 2011 |
| Ken09Tk.20/5 | Kenya | 2009 | *Ornithodoros* tick | HM745284 | HM745332 | HM745389 | X | L4.2.2.1 | Gallardo *et al.* 2011 |
| Kenya1950 | Kenya | 1950 | Domestic pig | AY261360 | EU874353 | EU874297 | X | L4.2.2.2.3 | Kutish *et al.* 2003 |
| ker64 | Kenya | 1964 | Domestic pig | AY578697 | - | - | I | L1.1 | Zsak *et al.* 2005 |
| KilleanI | Kenya | 1959 | *Phaecochoerus aethiopicus* | AY351550 | - | - | X | L4.2.2.2.2 | Lubisi *et al.* 2005 |
| KilleanII | Kenya | 1959 | *Phaecochoerus aethiopicus* | AY351551 | - | - | X | L4.2.2.2.3 | Lubisi *et al.* 2005 |
| KilleanIII | Kenya | 1959 | *Phaecochoerus aethiopicus* | AY351531 | - | - | X | L4.2.2.2.3 | Lubisi *et al.* 2005 |
| KimakiaI | Kenya | 1961 | *Potamochoerus porcus* | AY351533 | - | - | I | L1.1 | Lubisi *et al.* 2005 |
| KimakiaII | Kenya | 1961 | *Potamochoerus porcus* | AY351534 | - | - | I | L1.1 | Lubisi *et al.* 2005 |
| KIRT/89/2 | Tanzania | 1989 | *Ornithodoros* tick | AY351511 | - | - | I | L3.1 | Lubisi *et al.* 2005 |
| KIRT/89/3 | Tanzania | 1989 | *Ornithodoros* tick | AY351512 | - | - | X | L4.2.2.2.3 | Lubisi *et al.* 2005 |
| KIRT/89/4 | Tanzania | 1989 | *Ornithodoros* tick | AY351513 | - | - | X | L4.2.2.2.3 | Lubisi *et al.* 2005 |
| KIRW/89/1 | Tanzania | 1989 | *Phaecochoerus aethiopicus* | AY351514 | - | - | X | L4.2.2.2.3 | Lubisi *et al.* 2005 |
| KLI/88/2 | Zambia | 1988 | *Sus scrofa* | AY351553 | EU874347 | EU874258 | VIII | L3.1 | Lubisi *et al.* 2005 |
| kn66 | Kenya | 1966 | Domestic pig | AY578698 | - | - | X | L4.2.2.2.3 | Zsak *et al.* 2005 |
| Kwh/12 | Tanzania | 1968 | *Phaecochoerus* | AF301546 | - | - | X | L4.2.2.2.3 | Bastos *et al.* 2003 |
| LIL/89/1 | Malawi | 1989 | *Sus scrofa* | AY351505 | - | - | VIII | L3.1 | Lubisi *et al.* 2005 |
| LIL/90/1 | Malawi | 1990 | *Sus scrofa* | AY351510 | - | - | VIII | L3.1 | Lubisi *et al.* 2005 |
| Lillie | RSA | 1973 | *Ornithodoros* tick | DQ250109 | EU874341 | EU874306 | XX | L2.2.4 | Boshoff *et al.* 2007 |
| Lis57 | Portugal | 1957 | Domestic pig | AF301537 | FJ174420 | - | I | L1.1 | Bastos *et al.* 2003 |
| Lis60 | Portugal | 1960 | Domestic pig | AF301539 | X84889 | EU874273 | I | L1.1 | Bastos *et al.* 2003 |
| LIV/10/11 | Zambia | 1983 | *Ornithodoros* tick | AY351535 | - | - | I | L1.1.1 | Lubisi *et al.* 2005 |
| LIV/12/17 | Zambia | 1983 | *Ornithodoros* tick | AY351524 | - | - | I | L1.1.2 | Lubisi *et al.* 2005 |
| LIV/13/33 | Zambia | 1983 | *Ornithodoros* tick | AY494560 | - | - | I | L1.1 | Lubisi *et al.* 2005 |
| LIV/5/4 | Zambia | 1983 | *Ornithodoros* tick | AY351537 | - | - | I | L1.1.2 | Lubisi *et al.* 2005 |
| LIV/9/31 | Zambia | 1983 | *Ornithodoros* tick | AY351538 | - | - | I | L1.1.2 | Lubisi *et al.* 2005 |
| LIV/9/35 | Zambia | 1983 | *Ornithodoros* tick | AY351539 | - | - | I | L1.1.2 | Lubisi *et al.* 2005 |
| LIV5/40 | Zambia | 1982 | *Ornithodoros* tick | AY351536 | - | - | I | L1.1.2 | Lubisi *et al.* 2005 |
| LUS/93/1 | Zambia | 1991 | *Sus scrofa* | AY351563 | EU874377 | EU874275 | I | L1.2 | Lubisi *et al.* 2005 |
| M1 | RSA | 1966 | *Ornithodoros* tick | AY578699 | - | - | XIX | L2.2.3 | Zsak *et al.* 2005 |
| M61 | Spain | 1961 | Domestic pig | FJ174345 | FJ174385 | - | I | L1.1 | gallardo *et al.* 2009 |
| MAD/1/1998 | Madagascar | 1998 | Domestic pig | AF270706 | - | - | II | L1.2 | Bastos *et al.* 2003 |
| Madrid/62 | Spain | 1962 | NC | AF449461 | - | - | I | L1.1 | Bastos *et al.* 2003 |
| Mafra86 | Portugal | 1986 | Domestic pig | DQ028312 | DQ028321 | - | I | L1.1 | Duarte *et al.* 2005 |
| Mahaja02 | Madagascar | 2002 | Domestic pig | - | - | KC610549 | II | L1.2 | This study |
| MAL/2002/1 | Malawi | 2002 | *Sus scrofa* | AY494553 | EU874373 | EU874311 | V | L2.1.1 | Lubisi *et al.* 2005 |
| MAL/1978 | Malawi | 1978 |  | AF270707 | - | - | VIII | L3.1 | Bastos *et al.* 2003 |
| Malte78 | Malta | 1978 | Domestic pig | AF301543 | FJ174419 | - | I | L1.1 | Bastos *et al.* 2003 |
| MAN/89/2 | Zambia | 1989 | *Sus scrofa* | AY351562 | - | - | VIII | L3.1 | Lubisi *et al.* 2005 |
| Marovo02 | Madagascar | 2002 | Domestic pig | - | - | KC610550 | II | L1.2 | This study |
| MAU/2007/1 | Mauritius | 2007 | Domestic pig | FJ528594 | - | - | I | L1.2 | Lubisi *et al.* 2009 |
| MAU/2008/1 | Mauritius | 2008 | Domestic pig | FJ528595 | - | - | I | L1.2 | Lubisi *et al.* 2009 |
| MCH/89/1 | Malawi | 1989 | *Sus scrofa* | AY351506 | EU874352 | - | VIII | L3.1 | Lubisi *et al.* 2005 |
| MCH/89/3 | Malawi | 1989 | *Sus scrofa* | AY351507 | EU874351 | EU874259 | VIII | L3.1 | Lubisi *et al.* 2005 |
| Mchinji075 | Malawi | 1997 | *Sus scrofa* | AY351508 | - | - | VIII | L3.1 | Lubisi *et al.* 2005 |
| MFUE/6/1 | Zambia | 1982 | *Ornithodoros* tick | AY351561 | - | - | XII | L3.3 | Lubisi *et al.* 2005 |
| MHC/89/1 | Malawi | 1989 | Domestic pig | - | - | EU874292 | VIII | L3.1 | Heath *et al.* 2008 |
| MIMIVIRUS_CAPS1 | - | - | *Acanthamoeba polyphaga* | Q5UPL7 | - | - | - | - | pfam04451 |
| MIMIVIRUS_CAPS2 | - | - | *Acanthamoeba polyphaga* | Q7T6X4 | - | - | - | - | pfam04451 |
| MIMIVIRUS_CAPS4 | - | - | *Acanthamoeba polyphaga* | Q7T6Y5 | - | - | - | - | pfam04451 |
| Mkuzi78 | RSA | 1978 | *Ornithodoros* tick | AY578700 | - | - | I | L1.1.1 | Zsak *et al.* 2005 |
| MKUZI79 | RSA | 1979 | *Ornithodoros* tick | AY261362 | EU874367 | EU874294 | I | L1.1.1 | Kutish *et al.* 2003 |
| Morama98 | Madagascar | 1998 | Domestic pig | - | KC610533 | KC610551 | II | L1.2 | This study |
| Moronda02 | Madagascar | 2002 | Domestic pig | KC610520 | KC610532 | KC610552 | II | L1.2 | This study |
| MOZ/02/1 | Mozambique | 2002 | Domestic pig | - | EU874380 | EU874315 | II | L1.2 | Heath *et al.* 2008 |
| MOZ/02/2 | Mozambique | 2002 | Domestic pig | - | EU874376 | EU874274 | II | L1.2 | Heath *et al.* 2008 |
| MOZ/03/1 | Mozambique | 2003 | NC | - | - | EU874314 | II | L1.2 | Heath *et al.* 2008 |
| MOZ/05/1 | Mozambique | 2005 | NC | - | - | EU874313 | V/VI | L2.1 | Heath *et al.* 2008 |
| Moz/1/03 | Mozambique | 2003 | NC | FJ175199 | EU874379 | - | II | L1.2 | Heath *et al.* 2008 |
| MOZ/94/1 | Mozambique | 1994 | Domestic pig | AF270711 | - | - | VI | L2.1.2 | Bastos *et al.* 2003 |
| Moz/1/05 | Mozambique | 2005 | NC | FJ175200 | - | - | II | L1.2 | Heath *et al.* 2008 |
| MOZ/1/98 | Mozambique | 1998 | NC | AF270705 | - | - | VIII | L3.1 | Bastos *et al.* 2003 |
| MOZ/1960 | Mozambique | 1960 | Domestic pig | AF270708 | EU874371 | EU874309 | V | L2.1.1 | Bastos *et al.* 2004 |
| MOZ/1979 | Mozambique | 1979 | Domestic pig | AF270709 | EU874372 | EU874310 | V | L2.1.1 | Bastos *et al.* 2004 |
| MOZ/94/1 | Mozambique | 1994 | Domestic pig | - | EU874342 | EU874263 | V/VI | L2.1 | Heath *et al.* 2008 |
| MOZ/94/8 | Mozambique | 1994 | Domestic pig | - | EU874343 | EU874276 | V/VI | L2.1 | Bastos *et al.* 2003 |
| Moz/98/1 | Mozambique | 1998 | NC | - | EU874385 | EU874317 | VIII | L3.1 | Heath *et al.* 2008 |
| Moz/1/05 | Mozambique | 2005 | NC | - | EU874378 | - | - | L1 | Heath *et al.* 2008 |
| Moz64 | Mozambique | 1964 | Domestic pig | FJ174376 | FJ174422 | - | V | L2.1.1 | Gallardo *et al.* 2009 |
| MOZ/2001/1 | Mozambique | 2001 | *Sus scrofa* | AY351516 | - | - | VIII | L3.1 | Lubisi *et al.* 2005 |
| MOZ/2002/1 | Mozambique | 2002 | *Sus scrofa* | AY351517 | - | - | II | L1.2 | Lubisi *et al.* 2005 |
| MOZ/2002/2 | Mozambique | 2002 | *Sus scrofa* | AY351518 | - | - | II | L1.2 | Lubisi *et al.* 2005 |
| MOZ/60/98 | Mozambique | 1998 | *Sus scrofa* | AY274455 | - | - | II | L1.2 | Bastos *et al.* 2004 |
| MOZ/61/98 | Mozambique | 1998 | *Sus scrofa* | AY274456 | - | - | II | L1.2 | Bastos *et al.* 2004 |
| MOZ/62/98 | Mozambique | 1998 | *Sus scrofa* | AY274457 | - | - | VIII | L3.1 | Bastos *et al.* 2004 |
| MOZ/63/98 | Mozambique | 1998 | *Sus scrofa* | AY274458 | - | - | II | L1.2 | Bastos *et al.* 2004 |
| MOZ/70/98 | Mozambique | 1998 | *Sus scrofa* | AY274459 | - | - | II | L1.2 | Bastos *et al.* 2004 |
| MOZ/77/98 | Mozambique | 1998 | *Sus scrofa* | AY538726 | - | - | II | L1.2 | Bastos *et al.* 2004 |
| MOZ/94/8 | Mozambique | 1994 | Domestic pig | AF270712 | - | - | VI | L2.1.2 | Bastos *et al.* 2004 |
| MOZ/A/98 | Mozambique | 1998 | *Sus scrofa* | AY274452 | - | - | VIII | L3.1 | Bastos *et al.* 2004 |
| MOZ/B/98 | Mozambique | 1998 | *Sus scrofa* | AY274453 | - | - | VIII | L3.1 | Bastos *et al.* 2004 |
| MOZ/C/98 | Mozambique | 1998 | *Sus scrofa* | AY274454 | - | - | VIII | L3.1 | Bastos *et al.* 2004 |
| MPI/89/1 | Zambia | 1989 | *Sus scrofa* | AY351540 | - | - | VIII | L3.1 | Lubisi *et al.* 2005 |
| MPO89/1 | Zambia | 1989 | *Sus scrofa* | AY351541 | - | - | VIII | L3.1 | Lubisi *et al.* 2005 |
| MT325_M269L | - | - | *Chlorella sp*. strain Pbi | ABT13823 | - | - | - | - | pfam04451 |
| Mu82 | Spain | 1982 | Domestic pig | FJ174352 | FJ174395 | - | I | L1.1 | Gallardo *et al.* 2009 |
| MUR/07/1 | NC | 2007 | NC | - | EU874384 | EU874316 | II | L1.2 | Heath *et al.* 2008 |
| MWHOG/1 | Kenya | 1959 | *Phaecochoerus aethiopicus* | AY351548 | - | - | X | L4.2.2.2.3 | Lubisi *et al.* 2005 |
| MWHOG/3 | Kenya | 1959 | NC | AY351549 | - | - | X | L4.2.1 | Lubisi *et al.* 2005 |
| MWHOG/9 | Kenya | 1959 | *Phaecochoerus aethiopicus* | AY351565 | - | - | X | L4.2.2.1 | Lubisi *et al.* 2005 |
| MwLIL20/1 | Malawi | 1983 | *Ornithodoros* tick | L00966 | FJ174425 | - | VIII | L3.1 | Gallardo *et al.* 2009 |
| MZI/92/1 | Malawi | 1992 | *Sus scrofa* | AY351543 | - | EU874288 | XII | L3.3 | Lubisi *et al.* 2005 |
| MZI/94/1 | Malawi | 1994 | NC | - | EU874360 | - | - | L3 | Heath *et al.* 2008 |
| NAM/1/80 | Namibia | 1980 | *Phacochoerus* | AF504881 | - | - | I | L1.1 | Bastos *et al.* 2003 |
| NAM/1/95 | Namibia | 1995 | NC | DQ250122 | - | - | XVIII | L1.4 | Boshoff *et al.* 2007 |
| NDA/1/90 | Malawi | 1990 | Domestic pig | AF449473 | - | - | VIII | L3.1 | Bastos *et al.* 2003 |
| NGE/92/1 | Malawi | 1992 | *Sus scrofa* | AY351544 | - | - | VIII | L3.1 | Lubisi *et al.* 2005 |
| NH/P68 | Portugal | 1968 | Domestic pig | DQ028313 | DQ028322 | - | I | L1.1 | Duarte *et al.* 2005 |
| NIG/6 | Nigeria | 1998 | Domestic pig | AF270714 | - | - | I | L1.1 | Bastos *et al.* 2003 |
| NIG/1/99 | Nigeria | 1999 | Domestic pig | AF504887 | - | - | I | L1.1 | Bastos *et al.* 2003 |
| NIG/2/98 | Nigeria | 1998 | Domestic pig | AY972161 | - | - | I | L1.1 | Phologane *et al.* 2005 |
| NIG/3/98 | Nigeria | 1998 | Domestic pig | AY972162 | - | - | I | L1.1 | Phologane *et al.* 2005 |
| NIG/01/1 | Nigeria | 2001 | Domestic pig | - | EU874320 | EU874277 | I | L1.1 | Heath *et al.* 2008 |
| Nig01 | Nigeria | 2001 | Domestic pig | FJ174382 | FJ174426 | - | I | L1.1 | Gallardo *et al.* 2009 |
| NKZ88/1 | Zambia | 1988 | *Sus scrofa* | AY351554 | - | - | VIII | L3.1 | Lubisi *et al.* 2005 |
| Nu04.3 | Italy | 2004 | Domestic pig | FR668262 | FR668247 | - | I | L1.1 | Giammarioli *et al.* 2011 |
| Nu04.4 | Italy | 2004 | Domestic pig | FR668263 | FR668248 | - | I | L1.1 | Giammarioli *et al.* 2011 |
| Nu04.6a | Italy | 2004 | Domestic pig | FR668264 | FR668249 | - | I | L1.1 | Giammarioli *et al.* 2011 |
| Nu04.6b | Italy | 2004 | Domestic pig | FR668265 | FR668250 | - | I | L1.1 | Giammarioli *et al.* 2011 |
| Nu04WB | Italy | 2004 | Wild boar | FR668269 | FR668254 | - | I | L1.1 | Giammarioli *et al.* 2011 |
| Nu81 | Italy | 1981 | Domestic pig | FJ174358 | FJ174402 | - | I | L1.1 | Gallardo *et al.* 2009 |
| Nu90.1 | Italy | 1990 | Domestic pig | AF302813 | FJ174408 | - | I | L1.1 | Nix *et al.* 2006 |
| Nu91.3 | Italy | 1991 | Domestic pig | FJ174364 | FJ174409 | - | I | L1.1 | Gallardo *et al.* 2009 |
| Nu91.5 | Italy | 1991 | NC | FJ174365 | FJ174410 | - | I | L1.1 | Gallardo *et al.* 2009 |
| Nu93 | Italy | 1993 | Domestic pig | FJ174366 | FJ174411 | - | I | L1.1 | Gallardo *et al.* 2009 |
| Nu95.1 | Italy | 1995 | Domestic pig | FJ174368 | FJ174413 | - | I | L1.1 | Gallardo *et al.* 2009 |
| Nu96 | Italy | 1996 | Domestic pig | FJ174369 | FJ174414 | - | I | L1.1 | Gallardo *et al.* 2009 |
| Nu97 | Italy | 1997 | Domestic pig | FJ174370 | FJ174415 | - | I | L1.1 | Gallardo *et al.* 2009 |
| Nu98.3 | Italy | 1998 | Domestic pig | FJ174372 | FJ174417 | - | I | L1.1 | Gallardo *et al.* 2009 |
| Nu98.8B | Italy | 1998 | Domestic pig | FJ174373 | FJ174418 | - | I | L1.1 | Gallardo *et al.* 2009 |
| NUR/90/1 | Italy | 1990 | NC | AF302813 | - | - | I | L1.1 | Bastos *et al.* 2003 |
| NY2A_B059R | - | - | Chlorella | YP_001497255 | - | - | - | - | pfam04451 |
| NY2A_B529R | - | - | Chlorella | YP_001497725 | - | - | - | - | pfam04451 |
| NY2A_B585L | - | - | Chlorella | YP_001497781 | - | - | - | - | pfam04451 |
| NYA/1/2 | Zambia | 1986 | *Ornithodoros* tick | AY351555 | EU874330 | EU874302 | XIV | - | Lubisi *et al.* 2005 |
| o1 | RSA | 1996 | *Ornithodoros* tick | AY578701 | - | - | XIX | L2.2.3 | Zsak *et al.* 2005 |
| Ori84 | Italy | 1984 | Domestic pig | FJ174360 | FJ174404 | - | I | L1.1 | Nix *et al.* 2006 |
| Ori85 | Italy | 1985 | Domestic pig | FJ174361 | FJ174405 | - | I | L1.1 | Nix *et al.* 2006 |
| Ori90 | Italy | 1990 | Domestic pig | FJ174363 | FJ174407 | - | I | L1.1 | Nix *et al.* 2006 |
| Ori93 | Italy | 1993 | Domestic pig | FJ174367 | FJ174412 | - | I | L1.1 | Gallardo *et al.* 2009 |
| OURT88/1 | Portugal | 1988 | *Ornithodoros* tick | AF302811 | - | - | I | L1.1 | Bastos *et al.* 2003 |
| OURT88/3 | Portugal | 1988 | *Ornithodoros* tick | AM712240 | AM712240 | AM712240 | I | L1.1 | Nix *et al.* 2006 |
| PBCV1_A10R | - | - | *Chlorella* | Q89345 | - | - | - | - | pfam04451 |
| PBCV1_A11L | - | - | *Chlorella* | Q89346 | - | - | - | - | pfam04451 |
| PBCV1_A622L | - | - | *Chlorella* | O41104 | - | - | - | - | pfam04451 |
| PHW/88/1 | Zambia | 1988 | *Sus scrofa* | AY351567 | EU874366 | EU874257 | VIII | L3.1 | Lubisi *et al.* 2005 |
| PHYCO_ChainA | - | - | *Chlorella* | 1J5Q_A | - | - | - | - | pfam04451 |
| Portalegre90 | Portugal | 1990 | Domestic pig | DQ028314 | DQ028323 | - | I | L1.1 | Duarte *et al.* 2005 |
| POV01 | - | - | *Pyramimonas orientalis* | A7U6E9 | - | - | - | - | pfam04451 |
| PPV01 | - | - | *Phaeocystis pouchetii* | A7U6F0 | - | - | - | - | pfam04451 |
| Pr4 | RSA | 1996 | *Ornithodoros* tick | AY578702 | AY261363 | AY261363 | XX | L2.2.4 | Kutish *et al.* 2003 |
| Pr5 | RSA | 1996 | *Ornithodoros* tick | Q5IZK1 | - | - | - | - | Zsak *et al*., 2005 |
| RSA/03/1 | RSA | 2003 | NC | - | EU874333 | EU874278 | XX | L2.2.4 | Heath *et al.* 2008 |
| RSA/03/2 | RSA | 2003 | NC | - | EU874334 | EU874279 | III | L2.2.1 | Heath *et al.* 2008 |
| RSA/03/3 | RSA | 2003 | NC | - | EU874337 | EU874280 | III | L2.2.1 | Heath *et al.* 2008 |
| RSA/04/1 | RSA | 2004 | NC | - | EU874336 | EU874284 | III | L2.2.1 | Heath *et al.* 2008 |
| RSA/04/3 | RSA | 2004 | NC | - | EU874370 | EU874308 | IV | L2.2.2 | Heath *et al.* 2008 |
| RSA/07/1 | RSA | 2007 | NC | - | EU874383 | - | - | L2 | Heath *et al.* 2008 |
| RSA/1/98 | RSA | 1998 | Domestic pig | AF302818 | - | - | VII | L2.3.1 | Bastos *et al.* 2003 |
| RSA/1/99W | RSA | 1999 | *Phacochoerus* | AF449477 | EU874369 | EU874307 | IV | L2.2.2 | Bastos *et al.* 2003 |
| RSA/95/1 | RSA | 1995 | NC | DQ250123 | EU874340 | EU874266 | XX | L2.2.4 | Boshoff *et al.* 2007 |
| RSA/95/4 | RSA | 1995 | NC | - | EU874332 | EU874295 | XX | L2.2.4 | Heath *et al.* 2008 |
| RSA/95/5 | RSA | 1995 | NC | DQ250124 | EU874358 | EU874286 | III | L2.2.1 | Boshoff *et al.* 2007 |
| RSA/96/1 | RSA | 1996 | NC | DQ250125 | EU874339 | - | XXI | L2.3.3 | Boshoff *et al.* 2007 |
| RSA/96/2 | RSA | 1996 | NC | DQ250126 | EU874335 | EU874281 | XIX | L2.2.3 | Boshoff *et al.* 2007 |
| RSA/96/3 | RSA | 1996 | NC | DQ250127 | EU874375 | EU874283 | XIX | L2.2.3 | Boshoff *et al.* 2007 |
| RSA/98/1 | RSA | 1998 | Domestic pig | - | EU874374 | EU874312 | VII | L2.3.1 | Heath *et al.* 2008 |
| Sa88 | Spain | 1988 | Domestic pig | FJ174353 | FJ174398 | - | I | L1.1 | Gallardo *et al.* 2009 |
| SAL/92/1 | Malawi | 1992 | *Sus scrofa* | AY351546 | - | - | VIII | L3.1 | Lubisi *et al.* 2005 |
| Se88 | Spain | 1988 | Domestic pig | FJ174354 | FJ174397 | - | I | L1.1 | Gallardo *et al.* 2009 |
| SIY/91/2 | Malawi | 1991 | *Sus scrofa* | AY351566 | - | - | VIII | L3.1 | Lubisi *et al.* 2005 |
| SPEC/120 | RSA | 1987 | NC | AF302812 | - | - | XIX | L2.2.3 | Bastos *et al.* 2000 |
| SPEC/125 | RSA | 1987 | NC | DQ250112 | - | - | XIX | L2.2.3 | Boshoff *et al.* 2007 |
| SPEC/140 | RSA | 1987 | *Ornithodoros* tick | FJ455840 | - | - | III | L2.2.1 | Arnot *et al.* 2009 |
| SPEC/154 | Botswana | 1987 | NC | DQ250113 | EU874359 | EU874291 | VII | L2.3.1 | Boshoff *et al.* 2007 |
| SPEC/205 | Namibia | 1989 | NC | DQ250114 | EU874329 | EU874305 | I | L1.1.1 | Boshoff *et al.* 2007 |
| SPEC/207 | Namibia | 1989 | NC | DQ250115 | - | - | I | L1.1.1 | Boshoff *et al.* 2007 |
| SPEC/209 | Namibia | 1989 | NC | DQ250116 | EU874365 | EU874290 | I | L1.1.1 | Boshoff *et al.* 2007 |
| SPEC/245 | RSA | 1992 | NC | DQ250117 | EU874381 | - | XXII | L2.3.2 | Boshoff *et al.* 2007 |
| SPEC/251 | RSA | 1992 | NC | DQ250118 | - | - | XIX | L2.2.3 | Boshoff *et al.* 2007 |
| SPEC/257 | RSA | 1993 | NC | DQ250120 | EU874338 | EU874265 | III | L2.2.1 | Boshoff *et al.* 2007 |
| SPEC/260 | RSA | 1993 | NC | DQ250121 | - | - | VII | L2.3.1 | Boshoff *et al.* 2007 |
| SPEC/265 | Mozambique | 1994 | Domestic pig | AF270710 | EU874344 | EU874264 | VI | L2.1.2 | Bastos *et al.* 2003 |
| SPEC/53 | RSA | 1983 | NC | DQ250111 | - | - | XXI | L2.3.3 | Boshoff *et al.* 2007 |
| SPEC/57 | RSA | 1985 | *Ornithodoros* tick | FJ455839 | - | - | III | L2.2.1 | Arnot *et al.* 2009 |
| Ss04.10 | Italy | 2004 | Domestic pig | FR668266 | FR668251 | - | I | L1.1 | Giammarioli *et al.* 2011 |
| Ss05.3a | Italy | 2005 | Domestic pig | FR668267 | FR668252 | - | I | L1.1 | Giammarioli *et al.* 2011 |
| Ss05.3b | Italy | 2005 | Domestic pig | FR668268 | FR668253 | - | I | L1.1 | Giammarioli *et al.* 2011 |
| SS81 | Italy | 1981 | Domestic pig | FJ174359 | FJ174403 | - | I | L1.1 | Nix *et al.* 2006 |
| Ss88 | Italy | 1988 | Domestic pig | FJ174362 | FJ174406 | - | I | L1.1 | Nix *et al.* 2006 |
| SUM/14/11 | Zambia | 1983 | *Ornithodoros* tick | AY351542 | EU874357 | EU874287 | XIII | L3.4 | Lubisi *et al.* 2005 |
| TAN/01/1 | Tanzania | 2001 | *Sus scrofa* | AY494552 | EU874356 | EU874303 | XV | L3.5 | Lubisi *et al.* 2005 |
| TAN/02/3 | Tanzania | 2002 | NC | - | EU874355 | - | XVI | L3.6 | Heath *et al.* 2008 |
| TAN/03/1 | Tanzania | 2003 | *Sus scrofa* | AY494550 | EU874354 | EU874304 | XVI | L3.6 | Lubisi *et al.* 2005 |
| TAN/08/MABIBO | Tanzania | 2008 | Domestic pig | - | GQ410768 | - | - | L3.7 | Mazinso *et al.* 2011 |
| TAN/08/MAZIMBU | Tanzania | 2008 | Domestic pig | GQ410765 | GO410767 | - | XV | L3.7 | Mazinso *et al.* 2011 |
| TAN/08/TURIANI | Tanzania | 2008 | Domestic pig | - | GQ410771 | - | - | L3.7 | Mazinso *et al.* 2011 |
| TAN/03/2 | Tanzania | 2003 | *Sus scrofa* | AY494551 | - | EU874255 | XVI | L3.6 | Lubisi *et al.* 2005 |
| Tanzania/87 | Tanzania | 1987 | Domestic pig | - | X84891 | - | XV | L3.5 | Sun *et al.* 1995 |
| Tengani62 | Malawi | 1962 | Domestic pig | - | EU874318 | EU874296 | V/VI | L2.1 | Heath *et al.* 2008 |
| TEN/89/1 | Zambia | 1989 | *Sus scrofa* | AY351556 | - | - | VIII | L3.1 | Lubisi *et al.* 2005 |
| Tengani60 | Malawi | 1960 | *Phacochoerus* | AF301541 | - | - | V | L2.1.1 | Bastos *et al.* 2003 |
| THY/90/1 | Malawi | 1990 | *Sus scrofa* | AY351545 | - | - | VIII | L3.1 | Lubisi *et al.* 2005 |
| TMB/89/1 | Zambia | 1989 | *Sus scrofa* | AY351557 | EU874361 | EU874285 | VIII | L3.1 | Lubisi *et al.* 2005 |
| TNAVC | - | - | *Noctuidae* | Q4U3U9 | - | - | - | - | pfam04451 |
| Togo/98 | Togo | 1998 | NC | AF449481 | - | - | I | L1.1 | Bastos *et al.* 2003 |
| Tolagna01 | Madagascar | 2001 | Domestic pig | KC610519 | KC610531 | KC610553 | II | L1.2 | This study |
| Tolagna99 | Madagascar | 1999 | Domestic pig | KC610518 | EU620683 | EU620689 | II | L1.2 | This study |
| Toliar98 | Madagascar | 1998 | Domestic pig | - | KC610530 | KC610554 | II | L1.2 | This study |
| Trench | Kenya | 1959 | *Phaecochoerus aethiopicus* | AY351547 | - | - | X | L4.2.2.2.2 | Lubisi *et al.* 2005 |
| Tsididy08 | Madagascar | 2008 | Domestic pig | - | - | KC610555 | II | L1.2 | This study |
| Ug03H.1 | Uganda | 2003 | Domestic pig | FJ154428 | FJ174431 | - | IX | L4.1 | Gallardo *et al.* 2009 |
| Ug03H.2 | Uganda | 2003 | Domestic pig | FJ154429 | FJ174432 | - | IX | L4.1 | Gallardo *et al.* 2009 |
| Ug03H.3 | Uganda | 2003 | Domestic pig | FJ154430 | FJ174433 | - | IX | L4.1 | Gallardo *et al.* 2009 |
| Ug03P.4 | Uganda | 2003 | Domestic pig | FJ154431 | FJ174434 | - | IX | L4.1 | Gallardo *et al.* 2009 |
| Ug03P.5 | Uganda | 2003 | Domestic pig | FJ154432 | FJ174435 | - | IX | L4.1 | Gallardo *et al.* 2009 |
| Ug03P.6 | Uganda | 2003 | Domestic pig | FJ154433 | FJ174436 | - | IX | L4.1 | Gallardo *et al.* 2009 |
| UG07.F7 | Uganda | 2007 | Domestic pig | GQ477143 | GQ477150 | - | IX | L4.1 | Gallardo *et al.* 2009 |
| UG07.F8 | Uganda | 2007 | Domestic pig | GQ477144 | GQ477151 | - | IX | L4.1 | Gallardo *et al.* 2009 |
| UG07.Mukono | Uganda | 2007 | Domestic pig | GQ477142 | GQ477149 | - | IX | L4.1 | Gallardo *et al.* 2009 |
| UG07.Wak1 | Uganda | 2007 | Domestic pig | GQ477138 | GQ477145 | - | IX | L4.1 | Gallardo *et al.* 2009 |
| UG07.Wak2 | Uganda | 2007 | Domestic pig | GQ477139 | GQ477146 | - | IX | L4.1 | Gallardo *et al.* 2009 |
| UG07.Wak3 | Uganda | 2007 | Domestic pig | GQ477140 | GQ477147 | - | IX | L4.1 | Gallardo *et al.* 2009 |
| UG07.Wak4 | Uganda | 2007 | Domestic pig | GQ477141 | GQ477148 | - | IX | L4.1 | Gallardo *et al.* 2009 |
| Ug64 | Uganda | 1964 | Domestic pig | FJ174383 | FJ174430 | - | X | L4.2.2.2.2 | Gallardo *et al.* 2009 |
| UGA/95/1 | Uganda | 1995 | Domestic pig | - | EU874362 | EU874300 | IX | L4.1 | Heath *et al.* 2008 |
| UGA/95/3 | Uganda | 1995 | Domestic pig | AF449476 | - | - | X | L4.2.2.2.3 | Bastos *et al.* 2003 |
| UGA2003/1 | Uganda | 2003 | *Sus scrofa* | AY351564 | - | - | IX | L4.1 | Lubisi *et al.* 2005 |
| Uganda | Uganda | 1965 | Domestic pig | L27499 | - | - | X | L4.2.2.2.2 | Bastos *et al.* 2003 |
| UgH03 | Uganda | 2003 | Domestic pig | EF121429 | - | - | IX | L4.1 | Blanco *et al.* 2006 |
| UgP03 | Uganda | 2003 | Domestic pig | - | - | - | IX | L4.1 | CISA-INIA web site |
| Vaccinia | - | - | - | DQ121394 | - | - | - | - | Garcel *et al.*, 2007 |
| Val76 | Spain | 1976 | Domestic pig | AF449462 | - | - | I | L1.1 | Bastos *et al.* 2003 |
| vic | Zimbabwe | 1983 | Domestic pig | AY578705 | - | - | I | L1.1 | Zsak *et al.* 2005 |
| VICT/90/1 | Zimbabwe | 1990 | *Ornithodoros* tick | AF449474 | - | - | I | L1.1.1 | Bastos *et al.* 2003 |
| Warmbaths | RSA | 1987 | *Ornithodoros* tick | AY261365 | AY261365 | AY261365 | III | L2.2.1 | Kutish *et al.* 2003 |
| wart | Namibia | 1980 | *Phacochoerus* | AY578706 | - | - | IV | L2.2.2 | Zsak *et al.* 2005 |
| Warthog | Namibia | 1980 | *Phacochoerus* | AY261366 | AY261366 | AY261366 | IV | L2.2.2 | Kutish *et al.* 2003 |
| wb | RSA | 1987 | *Ornithodoros* tick | AY578707 | - | - | III | L2.2.1 | Zsak *et al.* 2005 |
| YEL/88/4 | Zambia | 1988 | *Sus scrofa* | AY351558 | - | - | VIII | L3.1 | Lubisi *et al.* 2005 |
| Z85 | Spain | 1985 | Domestic pig | AF449465 | FJ174396 | - | I | L1.1 | Bastos *et al.* 2003 |
| Za | RDC | 1987 | Domestic pig | AY578708 | - | - | XIX | L2.2.3 | Zsak *et al.* 2005 |
| ZAM/01/1 | Zambia | 2001 | *Sus scrofa* | AY494554 | - | - | I | L1.1.4 | Lubisi *et al.* 2005 |
| ZAM/02/1 | Zambia | 2002 | *Sus scrofa* | AY494559 | - | - | I | L1.1.4 | Lubisi *et al.* 2005 |
| ZAM/01/2 | Zambia | 2001 | *Sus scrofa* | AY494555 | - | - | I | L1.1.4 | Lubisi *et al.* 2005 |
| ZAM/01/3 | Zambia | 2001 | *Sus scrofa* | AY494556 | - | - | I | L1.1.4 | Lubisi *et al.* 2005 |
| ZAM/01/4 | Zambia | 2001 | *Sus scrofa* | AY494557 | - | - | I | L1.1.4 | Lubisi *et al.* 2005 |
| ZAM/01/5 | Zambia | 2001 | *Sus scrofa* | AY494558 | - | - | I | L1.1.4 | Lubisi *et al.* 2005 |
| ZAM/88/1 | Zambia | 1988 | *Sus scrofa* | AY351559 | - | - | VIII | L3.1 | Lubisi *et al.* 2005 |
| ZIM/92/1 | Zimbabwe | 1992 | NC | DQ250119 | EU874345 | - | XII | L1.3 | Boshoff *et al.* 2007 |
| ZON/88/1 | Zambia | 1988 | *Sus scrofa* | AY351560 | - | - | VIII | L3.1 | Lubisi *et al.* 2005 |

NC : not communicated
